# The Global, Regional, and National Burden of Psoriasis: Results and Insights From the Global Burden of Disease 2019 Study

**DOI:** 10.3389/fmed.2021.743180

**Published:** 2021-12-16

**Authors:** Giovanni Damiani, Nicola Luigi Bragazzi, Chante Karimkhani Aksut, Dongze Wu, Gianfranco Alicandro, Dennis McGonagle, Cui Guo, Robert Dellavalle, Ayman Grada, Priscilla Wong, Carlo La Vecchia, Lai-Shan Tam, Kevin D. Cooper, Mohsen Naghavi

**Affiliations:** ^1^Department of Dermatology, Case Western Reserve University, Cleveland, OH, United States; ^2^Clinical Dermatology, Istituto di Ricovero e Cura a Carattere Scientifico (IRCCS) Istituto Ortopedico Galeazzi, Milan, Italy; ^3^Department of Biomedical, Surgical and Dental Sciences, University of Milan, Milan, Italy; ^4^Chapel Allerton Hospital, University of Leeds, Leeds, United Kingdom; ^5^Department of Dermatology, University of Anschutz Medical Campus, Aurora, CO, United States; ^6^Department of Medicine and Therapeutics, The Prince of Wales Hospital, The Chinese University of Hong Kong, Shatin, Hong Kong SAR, China; ^7^Department of Clinical Sciences and Community Health, Università degli Studi di Milano, Milan, Italy; ^8^National Institute for Health Research (NIHR) Leeds Musculoskeletal Biomedical Research Unit, Section of Musculoskeletal Disease, Leeds Institute of Molecular Medicine, Chapel Allerton Hospital, University of Leeds, Leeds, United Kingdom; ^9^Jockey Club School of Public Health and Primary Care, The Chinese University of Hong Kong, Shatin, Hong Kong SAR, China; ^10^Department of Dermatology, Boston University School of Medicine, Boston, MA, United States; ^11^Institute for Health Metrics and Evaluation, University of Washington, Seattle, WA, United States

**Keywords:** psoriasis, prevalence, incidence, years lived with disability (YLDs), epidemiology, global health

## Abstract

**Background:** Psoriasis is a common, chronic, inflammatory, debilitating, systemic disease with a great impact on healthcare systems worldwide. As targeted therapies have transformed the therapeutic landscape, updated estimates of the Global Burden of Disease (GBD) imposed by psoriasis are necessary in order to evaluate the effects of past health care policies and to orient and inform new national and international healthcare strategies.

**Methods:** Data were extracted from the GBD 2019 study, which collates a systematic review of relevant scientific literature, national surveys, claims data, and primary care sources on the prevalence of psoriasis. Prevalence data were combined with disability weight (DW) to yield years lived with disability (YLDs). Measures of burden at global, regional, and national levels were generated for incidence, prevalence, and YLDs, due to psoriatic disease. All measures were reported as absolute numbers, percentages, and crude and age-adjusted rates per 100,000 persons. In addition, psoriasis burden was assessed by socio-demographic index (SDI).

**Findings:** According to the GBD 2019 methodology, there were 4,622,594 (95% uncertainty interval or UI 4,458,904–4,780,771) incident cases of psoriasis worldwide in 2019. The age-standardized incidence rate in 2019 was 57.8 (95% UI 55.8–59.7) per 100,000 people. With respect to 1990, this corresponded to a decrease of 20.0% (95% UI −20.2 to −19.8). By sex, the age-standardized incidence rate was similar between men [57.8 (95% UI 55.8–59.8) per 100,000 people] and women [(57.8 (95% UI 55.8–59.7) per 100,000 people]. With respect to 1990, this corresponded to a decrease by 19.5% (95% UI −19.8 to −19.2) and by 20.4% (95% UI −20.7 to −20.2) for men and women, respectively. The age-standardized incidence rate per 100,000 persons was found to vary widely across geographic locations. Regionally, high-income countries and territories had the highest age-standardized incidence rate of psoriasis [112.6 (95% UI 108.9–116.1)], followed by high-middle SDI countries [69.4 (95% UI 67.1–71.9)], while low SDI countries reported the lowest rate [38.1 (95% UI 36.8–39.5)]. Similar trends were detected for prevalence and YLDs.

**Conclusion:** In general, psoriasis burden is greatest in the age group of 60–69 years, with a relatively similar burden among men and women. The burden is disproportionately greater in high-income and high SDI index countries of North America and Europe. With advances in psoriasis therapeutics, objective evaluation of psoriasis disease burden is critical to track the progress at the population level.

## Introduction

Psoriatic disease is a complex, chronic, systemic, immune-mediated disease that represents a wide clinical spectrum ranging from cutaneous psoriasis to psoriatic arthritis, including dactylitis, enthesitis, psoriatic axial spondyloarthritis, and psoriatic onychopathy (1–4).

Epidemiological data on psoriatic disease are uncertain, with estimates of psoriasis prevalence ranging from 0.91 to 8.5% in adults and 0.0 to 2.1% in children (5). The global psoriasis prevalence rate is around 2–3% of the world population (6), reaching 8–11% in some Northern European countries (7). Remarkably, concerning the full spectrum of psoriatic disease, several observational studies pointed out that the proportion of undiagnosed psoriatic arthritis ranges from 10.9 to 29.0% in patients with psoriasis from European countries (8, 9), thereby suggesting that the real burden generated by psoriasis is significantly underestimated/under-reported. All the different manifestations of psoriatic disease share a similar pathogenetic, immunological (10, 11), and metabolic signature (12). Due to systemic inflammation, the psoriatic disease is often associated with other comorbidities that negatively impact social and private life, resulting in overall poor quality of life (13–15).

Furthermore, the increase in life expectancy, as well as the advent of targeted therapies and the improvement of healthcare services, could have increased the burden of the disease. Moreover, the dramatic demographic changes that occurred over the last four decades, including population growth and aging, could have impacted the burden of psoriatic disease as well; therefore, reliable, statistically robust, and updated estimates of psoriatic disease burden are necessary in order to evaluate the impact of past healthcare policies and, at the same time, to orient and inform new healthcare strategies in a data-driven, evidence-based fashion. Since the resolution by the World Health Assembly (WHA 67.9 2014), which aims to improve the healthcare and inclusion of people living with psoriasis, the Global Burden of Disease (GBD) initiative has increased its attention to the global epidemiology of the burden imposed by psoriasis, and the present study attempts to quantify it.

## Methods

### Overview of the Methodology

This study is part of the GBD 2019 (16), which, to the best of our knowledge, is the most comprehensive, methodologically robust report to date, which systematically estimates the spatial levels and temporal trends of the global burden caused by 369 diseases and injuries, as well as by 87 risk factors, in the period from 1990 to 2019. Seven super-regions, 21 regions, and 204 countries and territories were involved in the GBD 2019. The GBD 2019 adopts a 4-level hierarchical framework to classify and list causes as aggregate groupings. While level 1 causes include non-communicable disorders, injuries, and a category combining infectious, maternal, neonatal, and nutritional diseases/impairments, level 2 lists 22 diseases and injuries such as respiratory infections, cardiovascular disorders, and transport injuries. Level 3 and level 4 causes include specific causes, which differ based on the amount of details provided. For instance, psoriasis is a level 3 cause. Detailed GBD methodology is published elsewhere (16, 17).

Briefly, data on the disease burden attributable to psoriasis were extracted through a result tool on the website of the Institute for Health Metrics and Evaluation (IHME), University of Washington, Seattle, Washington, USA [http://ghdx.healthdata.org/gbd-results-tool]. The original data sources used for the estimations of the burden imposed by psoriasis can be found on the GBD 2019 Data Input Sources Tool website [http://ghdx.healthdata.org/gbd-2019/data-input-sources]. Since no identifiable data were used in the GBD 2019, a waiver of informed consent was in-depth reviewed and approved by the University of Washington Institutional Review Board (IRB).

### Definition

A brief overview specific to the psoriasis estimation strategy is presented in this study. Psoriasis was defined as an autoimmune disorder clinically characterized by areas of raised, red skin with silvery scales, which may be itchy. The pathogenesis and the precise mechanisms underlying the disease are complex and multi-factorial and yet to be fully elucidated. They include the immune-mediated activation of inflammatory pathways and cascades, resulting in the abnormal growth and behavior of certain types of skin cells. The case definition of psoriasis is based on the International Classification of Diseases (ICD)-10 codes, L40 and L41.

### Data Sources

The GBD 2019 has pooled together several input data obtained from four main sources, which include: (i) available scholarly literature; (ii) various large, nation-wide epidemiological surveys; (iii) claims data obtained from the United States, Taiwan, Poland, and Russia; and (iv) outpatient/primary care data from Norway.

Concerning the former data source, in the GBD 2010 study, a systematic review of the literature using an *ad hoc* devised search strategy had been carried out by the authors and collaborators of GBD 2010, using two major scholarly electronic databases (namely, PubMed/MEDLINE and Google Scholar) to retrieve and collect all relevant epidemiological data related to psoriasis (18, 19). This search was re-run and updated in the subsequent GBD 2013 and 2016 studies to capture all eligible studies published in the *interim* period (from 2012 to 2014 and from 2014 to 2016) (20, 21). Investigations were retained and included if (i) incidence or prevalence data of psoriasis were provided; (ii) samples representative of the general population (for instance, in order to avoid selection biases, subjects enrolled into experimental arms of randomized clinical trials or recruited in dermatological clinics were not considered) were utilized; (iii) large samples (i.e., sample size >100 participants) were utilized; and (iv) judged of high-quality in terms of methodology and study design.

Several epidemiological surveys were included: (i) the Medical Expenditure Panel Survey (MEPS) conducted in the United States between 2000 and 2009; (ii) the Australian National Health Survey (ANHS) carried out in several subsequent waves (from 1995 to 1996, in 2001, from 2004 to 2005, and from 2007 to 2008); and (iii) the USA National Health Nutrition Examination Survey (NHANES) conducted in 2002 and 2005.

Claims data obtained from the United States (until 2015–2016) and Taiwan, as well as from Poland (2015–2017) and Russia (2010–2017) were utilized, linking claims for multiple inpatients and/or outpatient encounters to single individuals. These were extracted as prevalent cases if having one or more inpatient/outpatient diagnosis encounters with a psoriasis-related ICD code. The Norwegian outpatient/primary care database had diagnoses linked to individuals, which were as such extracted as prevalent cases.

In summary, 8 sources were utilized for calculating psoriasis incidence (from four contributing countries) and 123 sources for computing psoriasis prevalence (from 31 contributing countries), whereas 15 additional sources (from one contributing country) were used for quantitatively assessing the proportion of psoriasis cases and computing the prevalence rate. Overall, 132 unique data sources were employed in the present investigation (from 31 contributing countries). The detailed data sources used to estimate and compute the burden of psoriasis in the different countries can be found by accessing the GBD 2019 Data Input Sources Tool at the following link: http://ghdx.healthdata.org/gbd-2019/data-input-sources5.

### Data Inclusion and Exclusion

According to the GBD methodology, outpatient data from healthcare settings based in the USA and Sweden were potentially eligible. However, after a thorough assessment, they could not be retained in the present investigation due to violations of well-consolidated regional patterns and age-related distribution trends. These violations could not be observed for other data sources from other countries as well, such as Norway.

Retained data were subsequently re-evaluated in terms of the presence of outliers (i.e., high values in young age groups), the inclusion of which would have resulted in a poor model fit or significant distortion/over-estimation of sub-national pseudo-random effects. Moreover, data found to be incoherent when compared to regional, super-regional, and global rate trends were excluded. None of the latter violations could be detected when data quality assessment was performed.

### Statistical Analysis

Several health metrics indicators were computed, including prevalence, incidence, and disability-related estimates. More in detail, incidence and prevalence data related to diagnoses ascertained as psoriasis cases and extracted from the previously described data sources (administrative databases, physical examination-based studies) were used as input and entered into Disease Modeling—Meta-regression (DisMod-MR) 2.1, a Bayesian meta-regression tool, to estimate epidemiological metrics by age [23 age groups: (i) early neonatal, (ii) late neonatal, (iii) post-neonatal, (iv) 1–4, (v) 5–9, (vi) 10–14, (vii) 15–19, (viii) 20–24, (ix) 25–29, (x) 30–34, (xi) 35–39, (xii) 40–44, (xiii) 45–49, (xiv) 50–54, (xv) 55–59, (xvi) 60–64, (xvii) 65–69, (xviii) 70–74, (xix) 75–79, (xx) 8–84, (xxi) 8–89, (xxii) 90–94, and (xxiii) 95+ years], sex (male, female, and male/female combined), year (from 1990 to 2019), and geography (in terms of super-regions, regions, countries, and territories).

Instead of DisMod-MR, another biostatistical approach termed as Meta-Regression—Bayesian Regularised Trimmed (MR-BRT) was utilized to process the USA Marketscan data, along with rheumatoid arthritis diagnosis extracted from administrative data, adjusting them toward the level of other prevalence datapoints, which were deemed to be more representative of the general population. Data related to rheumatoid arthritis were also extracted since the differential diagnosis of psoriatic disease includes rheumatoid arthritis.

Concerning modeling strategy, psoriasis remission and duration ranges were set at 0.05–0.15, and 6.6–20 years, respectively, based on the current knowledge of psoriasis-related epidemiology, the consensus of expert opinions, the existing scientific literature, and previously published GBD studies. Excess mortality due to psoriasis was measured in years of life lost and assumed to be zero. Data collated and compiled generated a database large enough to ensure the possibility of utilizing relatively short time spans (10-year windows) to compute the goodness-of-fit of the datapoints.

Study-level covariates (including the main features of the populations under study, such as age or gender) were utilized in order to mark data extracted from self-report, outpatient/primary care, and claims data. From the MR-BRT cross-walk adjustment analysis, setting the gamma parameter at 0.63, the beta coefficient (logit) for studies without physical examination was computed at −0.12 (ranging from −1.36 to 1.12), for studies utilizing the USA Marketscan 2000 data at −1.23 (ranging from −2.50 to −0.01), for studies employing the USA Marketscan 2010–2016 data at −0.82 (−2.06 to 0.43), and for studies with rheumatoid arthritis (RA) diagnosis obtained from administrative data at −0.87 (ranging from −2.12 to 0.37). The corresponding related adjustment factors yielded 0.47, 0.22, 0.31, and 0.29, respectively.

Socio-demographic index (SDI) and the absolute value of average latitude served as location-level (country-level) covariates to inform the estimation of the variables for countries and territories with the dearth of data. Exponentiated beta values (which can be understood as odds ratios, ORs) were computed at 0.19 (ranging from 0.17 to 0.20) and 1.01 (ranging from 1.01 to 1.01) for SDI and the absolute value of average latitude, respectively.

Prevalence estimates were multiplied by a multiplier known as disability weight (DW), computed from population-wide epidemiological surveys and an open-access web-based study, to yield years lived with disability (YLDs) and disability-adjusted life years (DALYs). The latter indicator combines in one measure the time lived with disability and the time lost due to premature mortality. A severity split analysis with DWs was conducted, according to the type and extent of *sequelae* (assessed as functional consequences and symptoms of the disease stage) (22). In other words, as done in previous GBD studies, different DWs for psoriasis were assigned based on its degree of disfigurement with itch/pain (levels of severity 1, 2, and 3) (23).

In the case of mild psoriasis, the subject reports a slight, even though visible physical deformity, which can be sore and/or itchy, besides causing psychological discomfort and worries. In this case, DW is 0.027 (ranging from 0.015 to 0.042). In the case of severe psoriasis, the individual has an obvious, very painful, itchy physical deformity, which causes psychological discomfort, worries, poor sleep quality, avoidance of social contacts, and suicidal thoughts. In this case, DW is 0.576 (ranging from 0.401 to 0.731). The intermediate case of moderate psoriasis is characterized by impaired sleep and concentration issues. In this case, DW is 0.188 (ranging from 0.124 to 0.267).

Measures of burden at the global, regional, and national levels were generated and estimated, both for epidemiological (incidence and prevalence) and disability (YLDs and DALYs) indicators due to psoriatic disease. All measures were reported as absolute (counts) and relative (percentages) numbers, both as crude and age-adjusted rates per 100,000 persons, where the procedure of age-standardization was applied based on the WHO world population age structure. All estimates were reported with their 95% uncertainty intervals (95% UI). These intervals were estimated by taking 1,000 samples from the posterior distribution of each quantity and using the 25th- and 97.5th-ordered draws of the uncertainty distribution. UIs are different from “classical” CIs, in enabling to capture and model uncertainty from multiple steps (such as model estimating, and parameter specifying steps), incorporating several, and also highly heterogeneous data sources. This is a considerable methodological advancement that ensures estimate robustness and reliability, with respect to “conventional” techniques that rely on sampling error alone.

Epidemiological and disability indicator estimates are also presented stratified according to the location/country level. Within the GBD methodological framework, countries are classified based on an objective measurement of their developmental status, namely, the SDI. This is a composite metric, which combines and summarizes various variables, including average income, educational attainment, and total fertility rate (TFR) under 25 years of age. Based on this computation, SDI is calculated and assigned to each country (24). SDI is scaled from zero, which represents the lowest income, educational attainment, and the highest TFR possible, to one, which, on the contrary, represents the highest income, educational achievement, and the lowest TFR possible. The relationship between epidemiological and disability rates and SDI status (categorized as high, high-middle, middle, low-middle, and low SDI countries) was conducted and is presented here.

Our present study reports findings in compliance with the Guidelines for Accurate and Transparent Health Estimates Reporting (GATHER) statement (25).

## Results

### Incidence of Psoriasis in 2019 and Its Spatio-Temporal Trend

**Figure 1:**
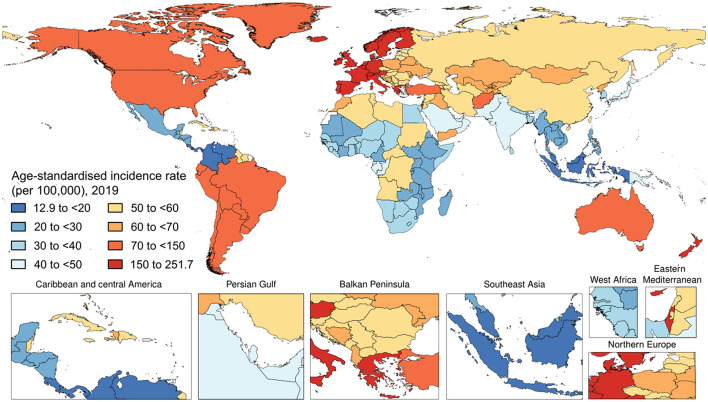
The national age-standardized incidence rate of psoriasis (per 100,000) in 2019.

Worldwide, there were 4,622,594 (95% UI 4,458,904–4,780,771) incident cases of psoriasis in 2019. The age-standardized incidence rate in 2019 was 57.8 (95% UI 55.8–59.7) per 100,000 people. With respect to 1990, this corresponded to a decrease by 20.0% (95% UI −20.2 to −19.8). By sex, the age-standardized incidence rate was similar between men [57.8 (95% UI 55.8–59.8) per 100,000 people] and females [57.8 (95% UI 55.8–59.7) per 100,000 people]. With respect to 1990, this corresponded to a decrease by 19.5% (95% UI −19.8 to −19.2) and 20.4% (95% UI −20.7 to −20.2), respectively. Figure 1 and Table 1 show the age-specific numbers and rates of incident psoriasis cases at the global level and stratified by sex and GBD region in 2019. As it can be seen, the age-standardized incidence rate per 100,000 persons was found to vary widely across geographic locations. Regionally, high-income countries and territories had the highest age-standardized incidence rate of psoriasis [112.6 (95% UI 108.9–116.1)], followed by high-middle SDI countries [69.4 (95% UI 67.1–71.9)], while low SDI countries reported the lowest rate [38.1 (95% UI 36.8–39.5)]. Low-middle SDI countries [(45.1 (95% UI 43.4–46.6)] and middle-SDI countries [41.7 (95% UI 40.2–43.1)] reported a similar burden. Middle SDI countries documented the highest change in the age-standardized incidence rate in 2019 with respect to 1990, with a decrease by 21.8% (95% UI −22.1 to −21.4), whereas high SDI countries reported the lowest change with a decrease by 10.2% (95% UI −10.6 to −9.7). In terms of GBD regions, the age-standardized incidence rate was highest in Western Europe [204.5 (95% UI 197.6–211.4)], followed by Australasia [145.4 (95% UI 139.6–151.4)] and high-income North America [92.7 (95% UI 89.7–95.5)], whereas it was lowest in Southeast Asia [20.1 (95% UI 19.3–20.8)], followed by central Latin America [20.7 (95% UI 19.9–21.5)] and Eastern Sub-Saharan Africa [25.1 (95% UI 24.2–26.1)]. The GBD region which reported the highest decrease in the age-standardized incidence rate was North Africa and the Middle East, with a decrease by 23.1% (95% UI −23.7 to −22.5) in 2019 with respect to 1990. The lowest change was documented in the high-income Asia Pacific, with a decrease of 3.4% (95% UI −4.0 to −2.7). At the national level, the country with the highest rate was France [251.7 (95% UI 242.5–261.0)], whereas the lowest rate was reported in Indonesia [12.9 (95% UI 12.4–13.4)]. For further details, the reader can refer to Supplementary Table 1.

**Table 1:** Incidence, prevalence, and YLDs of psoriasis in 2019 and percentage change of age-standardized rates, by sex and GBD region.

	**Incidence**			**Prevalence**			**YLDs**		
	**Number**	**Age-standardised rate (per 100 000 people)**	**Percentage change in age-standardised rates, 1990–2019**	**Number**	**Age-standardised rate (per 100 000 people)**	**Percentage change in age-standardised rates, 1990–2019**	**Number**	**Age-standardised rate (per 100 000 people)**	**Percentage change in age-standardised rates, 1990–2019**
Global	4,622,594 (4,458,904 to 4,780,771)	57.8 (55.8 to 59.7)	−20.0% (−20.2 to −19.8)	40,805,386 (39,421,384 to 42,076,746)	503.6 (486.9 to 519.2)	−23.7% (−24.0 to −23.5)	3,505,736 (2,504,956 to 4,638,757)	43.3 (30.9 to 57.4)	−23.6% (−24.2 to −23.0)
**Sex**									
Male	2,315,841 (2,232,986 to 2,395,283)	57.8 (55.8 to 59.8)	−19.5% (−19.8 to −19.2)	20,448,885 (19,749,817 to 21,083,370)	510.7 (493.4 to 526.4)	−22.9% (−23.3 to −22.6)	1,772,054 (1,261,969 to 2,355,815)	44.2 (31.5 to 58.7)	−22.8% (−23.7 to −22.0)
Female	2,306,753 (2,224,308 to 2,384,894)	57.8 (55.8 to 59.7)	−20.4% (−20.7 to −20.2)	20,356,501 (19,672,490 to 20,990,702)	497.5 (481.1 to 513.1)	−24.4% (−24.6 to −24.1)	1,733,683 (1,241,898 to 2,282,839)	42.5 (30.4 to 56.1)	−24.2% (−25.1 to −23.4)
**GBD region**									
Central Sub-Saharan Africa	61,984 (59,320 to 64,656)	51.6 (49.4 to 53.8)	−16.5% (−17.8 to −15.0)	445,748 (427,754 to 462,996)	412.7 (397.2 to 427.9)	−19.1% (−20.1 to −18.0)	39,139 (27,548 to 52,169)	35.6 (25.0 to 47.1)	−18.2% (−23.9 to −12.4)
Eastern Sub-Saharan Africa	89,695 (86,383 to 93,561)	25.1 (24.2 to 26.1)	−9.8% (−10.3 to −9.2)	551,213 (530,511 to 573,329)	166.8 (160.8 to 172.9)	−9.4% (−10.0 to −8.9)	48,807 (34,514 to 65,163)	14.5 (10.3 to 19.2)	−8.8% (−12.7 to −5.2)
Southern Sub-Saharan Africa	25,357 (24,396 to 26,331)	32.9 (31.7 to 34.1)	−10.6% (−11.3 to −10.0)	168,145 (162,227 to 174,017)	223.0 (215.2 to 230.2)	−10.5% (−11.0 to −9.8)	14,553 (10,384 to 19,199)	19.2 (13.7 to 25.3)	−11.0% (−15.2 to −6.6)
Western Sub-Saharan Africa	131,825 (127,072 to 137,224)	32.5 (31.3 to 33.6)	−20.2% (−20.5 to −19.8)	840,876 (810,132 to 871,926)	225.2 (217.3 to 232.9)	−23.1% (−23.5 to −22.7)	74,188 (52,193 to 98,677)	19.5 (13.9 to 25.9)	−22.7% (−24.9 to −20.7)
Andean Latin America	52,103 (49,796 to 54,275)	82.4 (78.8 to 85.8)	−13.1% (−14.3 to −11.9)	444,522 (427,695 to 461,805)	712.9 (685.9 to 739.6)	−16.3% (−17.3 to −15.2)	38,808 (27,362 to 51,290)	62.1 (44.0 to 82.0)	−16.2% (−20.2 to −11.9)
Tropical Latin America	202,572 (195,312 to 209,349)	87.1 (84.1 to 90.0)	−5.7% (−6.2 to −5.1)	1,830,253 (1,765,446 to 1,893,664)	767.2 (741.1 to 792.9)	−5.2% (−5.7 to −4.6)	157,656 (112,010 to 207,987)	66.2 (46.9 to 87.6)	−4.6% (−6.9 to −2.5)
Central Latin America	52,370 (50,378 to 54,362)	20.7 (19.9 to 21.5)	−12.2% (−12.7 to −11.7)	334,104 (322,899 to 345,848)	132.0 (127.7 to 136.5)	−12.3% (−12.8 to −11.8)	29,285 (20,556 to 39,034)	11.6 (8.1 to 15.4)	−11.9% (−15.4 to −8.3)
Southern Latin America	70,211 (67,264 to 73,298)	102.1 (97.9 to 106.3)	−7.3% (−8.8 to −5.7)	650,187 (624,126 to 674,258)	898.7 (863.3 to 933.3)	−12.9% (−14.1 to −11.7)	56,128 (39,341 to 74,462)	78.0 (54.5 to 103.2)	−12.9% (−17.3 to −8.1)
Caribbean	27,467 (26,394 to 28,614)	56.9 (54.7 to 59.2)	−7.1% (−8.1 to −6.1)	202,492 (195,171 to 209,683)	413.1 (398.4 to 427.7)	−7.5% (−8.3 to −6.6)	17,529 (12,493 to 23,481)	35.8 (25.5 to 47.9)	−7.6% (−12.2 to −2.9)
Central Europe	76,996 (74,396 to 79,469)	60.3 (58.5 to 62.2)	−15.5% (−16.3 to −14.6)	624,819 (606,034 to 641,226)	440.8 (428.2 to 452.1)	−18.1% (−19.0 to −17.2)	53,356 (38,034 to 70,323)	38.3 (27.0 to 50.8)	−17.8% (−20.2 to −15.5)
Eastern Europe	139,976 (134,745 to 144,910)	59.5 (57.6 to 61.5)	−11.3% (−11.9 to −10.6)	1,072,655 (1,036,778 to 1,105,301)	423.7 (410.5 to 436.8)	−13.0% (−13.7 to −12.2)	91,625 (64,980 to 120,358)	36.7 (25.8 to 48.4)	−12.5% (−15.0 to −10.2)
North Africa and Middle East	336,787 (324,072 to 349,591)	55.5 (53.5 to 57.5)	−23.1% (−23.7 to −22.5)	2,426,320 (2,342,733 to 2,509,856)	414.3 (400.3 to 427.8)	−26.9% (−27.5 to −26.2)	211,107 (149,579 to 279,840)	35.8 (25.5 to 47.3)	−26.9% (−29.0 to −24.6)
Central Asia	58,976 (56,469 to 61,360)	62.2 (59.7 to 64.7)	−16.0% (−16.9 to −15.0)	420,455 (404,188 to 436,205)	454.9 (437.6 to 471.2)	−19.9% (−20.6 to −19.2)	36,746 (25,998 to 49,012)	39.6 (27.9 to 52.8)	−19.8% (−23.2 to −16.3)
South Asia	792,848 (765,638 to 821,309)	44.4 (42.9 to 46.0)	−8.2% (−8.6 to −7.8)	5,873,201 (5,668,229 to 6,075,556)	334.5 (322.9 to 345.6)	−5.1% (−5.6 to −4.7)	507,805 (359,434 to 669,841)	28.7 (20.4 to 37.8)	−4.6% (−6.8 to −2.1)
Southeast Asia	139,036 (133,404 to 144,184)	20.1 (19.3 to 20.8)	−12.9% (−13.4 to −12.4)	887,599 (856,929 to 918,713)	128.8 (124.5 to 133.2)	−14.3% (−14.8 to −13.7)	77,728 (55,264 to 104,730)	11.2 (8.0 to 15.2)	−13.8% (−17.2 to −10.2)
East Asia	925,967 (891,325 to 958,216)	54.1 (52.2 to 55.9)	−20.8% (−21.3 to −20.4)	7,948,327 (7,669,027 to 8,215,035)	436.6 (422.0 to 450.1)	−24.7% (−25.1 to −24.2)	688,337 (490,650 to 909,286)	38.1 (27.1 to 50.2)	−24.4% (−26.0 to −22.8)
Oceania	4,694 (4,512 to 4,889)	39.2 (37.6 to 40.8)	−9.1% (−10.5 to −7.7)	31,161 (29,970 to 32,394)	278.0 (267.9 to 288.3)	−9.2% (−10.5 to −7.8)	2,711 (1,891 to 3,664)	23.8 (16.7 to 31.9)	−9.3% (−16.9 to −0.7)
High-Income Asia Pacific	83,991 (80,923 to 86,862)	40.0 (38.5 to 41.4)	−3.4% (−4.0 to −2.7)	613,404 (591,525 to 633,631)	262.2 (253.4 to 270.8)	−4.3% (−5.0 to −3.6)	52,711 (37,862 to 69,195)	23.0 (16.4 to 30.7)	−4.1% (−8.4 to 0.1)
High-Income North America	359,271 (347,584 to 370,502)	92.7 (89.7 to 95.5)	−11.8% (−12.5 to −11.0)	4,693,639 (4,553,019 to 4,837,451)	1,081.6 (1,048.9 to 1,115.4)	−14.9% (−15.8 to −14.0)	392,467 (282,948 to 516,020)	92.0 (66.2 to 121.5)	−15.3% (−16.8 to −13.7)
Western Europe	946,916 (913,064 to 979530)	204.5 (197.6 to 211.4)	−5.7% (−6.2 to −5.2)	10,236,919 (9,862,029 to 10,589,120)	1,884.1 (1,817.4 to 1,948.3)	−9.4% (−10.0 to −8.9)	871,673 (623,279 to 1,153,354)	163.1 (115.8 to 216.7)	−9.4% (−10.7 to −8.1)
Australasia	43,553 (41,826 to 45,339)	145.4 (139.6 to 151.4)	−7.9% (−9.5 to −6.3)	509,347 (490,055 to 528,245)	1,506.1 (1,448.9 to 1,560.8)	−12.3% (−13.7 to −10.9)	43,378 (30,992 to 57,275)	129.9 (92.1 to 171.1)	−12.3% (−16.6 to −8.0)

*Data in parentheses are 95% uncertainty intervals (UI). GBD, global burden of disease, injuries, and risk factors study; YLDs, years lived with disability*.

### Prevalence of Psoriasis in 2019 and Its Spatio-Temporal Trend

**Figure 2:**
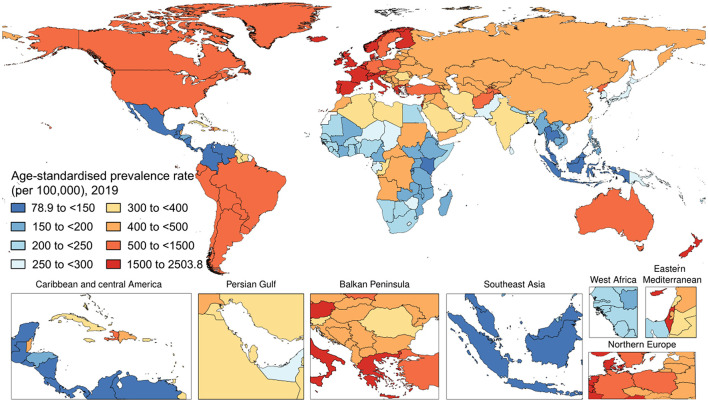
The national age-standardized prevalence rate of psoriasis (per 100,000) in 2019.

Worldwide, there were 40,805,386 (95% UI 39,421,384–42,076,746) prevalent cases of psoriasis in 2019 (Figure 2). The age-standardized prevalence rate in 2019 was 503.6 (95% UI 486.9–519.2) per 100,000 people. With respect to 1990, this corresponded to a decrease of 23.7% (95% UI −24.0 to −23.5). By sex, the age-standardized prevalence rate was similar between men [510.7 (95% UI 493.4–526.4) per 100,000 people] and women [497.5 (95% UI 481.1–513.1) per 100,000 people]. With respect to 1990, this corresponded to a decrease by 22.9% (95% UI −23.3 to −22.6) and by 24.4% (95% UI −24.6 to −24.1), respectively. Table 1 shows the age-specific numbers and rates of prevalent psoriasis cases at the global level and stratified by sex and GBD region in 2019. As it can be seen, the age-standardized prevalence rate per 100,000 persons was found to vary widely across geographic locations. Regionally, high-income countries and territories had the highest age-standardized prevalence rate of psoriasis [1,072.7 (95% UI 1,038.7–1,106.0)], followed by high-middle SDI countries [589.9 (95% UI 569.2–608.5)], while low SDI countries reported the lowest rate [300.8 (95% UI 290.5–311.1)]. Low-middle SDI countries [352.1 (95% UI 340.3–363.7)] and middle SDI countries [338.6 (95% UI 327.5–348.9)] reported a similar epidemiological burden. The most important temporal changes occurred in high-middle SDI countries [−21.1% (95% UI −21.4 to −20.7)] and middle SDI countries [−20.6% (95% UI −21.0 to −20.2)], whereas the lowest change was documented in low SDI countries [−11.1% (95% UI −11.5 to −10.6)]. In terms of GBD regions, the age-standardized prevalence rate was highest in Western Europe [1,884.1 (95% UI 1,817.4–1,948.3)], followed by Australasia [95% UI 1,506.1 (1,448.9–1,560.8)] and high-income North America [1,081.6 (95% UI 1,048.9–1,115.4)], whereas they were lowest in Southeast Asia [128.8 (95% UI 124.5–133.2)], followed by central Latin America [132.0 (95% UI 127.7–136.5)] and Eastern Sub-Saharan Africa [166.8 (95% UI 160.8–172.9)]. The GBD region which reported the highest decrease in the age-standardized prevalence rate was North Africa and the Middle East with a decrease of 26.9% (95% UI −27.5 to −26.2) in 2019 with respect to 1990. The lowest change was documented in the high-income Asia Pacific, with a decrease of 4.3% (95% UI −5.0 to −3.6). At the national level, the country with the highest rate was France [2,503.8 (2,395.4 to 2,608.6)], whereas the lowest rate was reported in Indonesia [79.8 (95% UI 76.9–82.7)]. For further details, the reader can refer to Supplementary Table 2.

### YLD of Psoriasis in 2019 and Its Spatio-Temporal Trend

**Figure 3:**
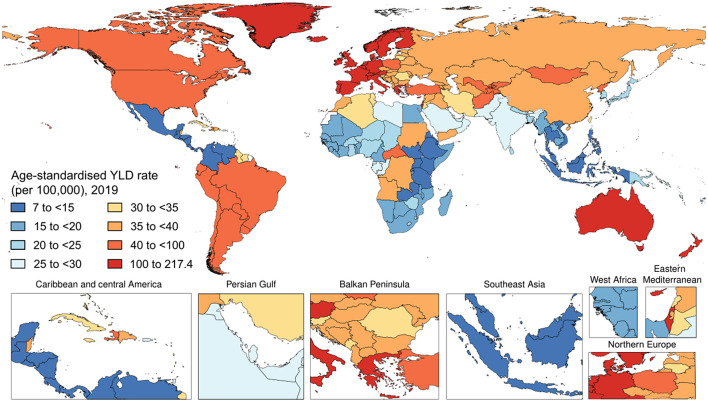
National age-standardized years lived with disability (YLDs) rate of psoriasis (per 100,000) in 2019.

Worldwide, psoriasis generated 3,505,736 (95% UI 2,504,956–4,638,757) YLD cases of psoriasis in 2019. The age-standardized YLD rate in 2019 was 43.3 (95% UI 30.9–57.4) per 100,000 people (Figure 3). With respect to 1990, this corresponded to a decrease by 23.6% (95% UI −24.2 to −23.0). By sex, the age-standardized YLD rate was similar between men [44.2 (95% UI 31.5–58.7) per 100,000 people] and women [42.5 (95% UI 30.4–56.1) per 100,000 people]. With respect to 1990, this corresponded to a decrease by 22.8% (95% UI −23.7 to −22.0) and by 24.2% (95% UI −25.1 to −23.4), respectively. Table 1 shows the age-specific numbers and rates of YLD psoriasis cases at the global level and stratified by sex and GBD region in 2019. As it can be seen, the age-standardized YLD rate per 100,000 persons was found to vary widely across geographic locations. Regionally, high-middle SDI countries [51.1 (95% UI 36.5–67.7)] and high SDI countries [92.3 (95% UI 65.6–122.2)] reported the highest age-standardized YLD rates, whereas low SDI countries [25.9 (95% UI 18.2–34.0)], low-middle SDI countries [30.3 (95% UI 21.6–40.2)], and middle SDI countries [29.4 (95% UI 20.8–38.8)] reported comparable rates for both sexes combined and also when stratifying according to gender, as pictorially shown in Supplementary Figure 1. The most important temporal changes occurred in high-middle SDI countries [−20.8% (95% UI −21.9 to −19.6)] and middle SDI countries [-20.5% (95% UI −21.9 to −19.1)], whereas the lowest change was documented in low SDI countries [-10.6% (95% UI −12.9 to −8.2)]. In terms of GBD regions, the age-standardized YLD rate was highest in Western Europe [163.1 (95% UI 115.8–216.7)], followed by Australasia [129.9 (95% UI 92.1–171.1)] and high-income North America [92.0 (95% UI 66.2–121.5)], whereas it was lowest in Southeast Asia [11.2 (95% UI 8.0–15.2)], followed by central Latin America [11.6 (95% UI 8.1–15.4)] and Eastern Sub-Saharan Africa [14.5 (95% UI 10.3–19.2)]. The GBD region which reported the highest decrease in the age-standardized prevalence rate was North Africa and the Middle East with a decrease of 26.9% (95% UI −29.0 to −24.6) in 2019 with respect to 1990. The lowest change was documented in the high-income Asia Pacific, with a decrease by −4.1% (95% UI −8.4 to 0.1). For further details, the reader can refer to Supplementary Table 3. Furthermore, the age-standardized YLD rate per 100,000 persons was found to gradually increase in the groups from 1–4 to 60–64 years, reaching its plateau in the 65–69 years group, and decreasing afterward, as pictorially shown in Supplementary Figures 2–4. In terms of YLDs, among level 3 causes, psoriasis was ranked as the 49th cause for both sexes combined, while it was ranked 50th and 52nd in 2010 and 2019, respectively.

## Discussion

The most recent iteration of the global disease burden estimation, GBD 2019, reveals that psoriasis burden is greatest in the age groups of 60–64 and 65–69 years, with relatively similar burden among males and females throughout all age groups. The high-income GBD super-region, specifically North America, Western Europe, Australasia, and Southern Latin America, shared the greatest incidence, prevalence, and DALY rates from psoriasis compared to the other world regions. Congruently, high SDI countries shared a greater psoriasis-registered burden compared to low or middle SDI countries. Our results indicate that the burden of psoriasis has varied little over the past 29 years. Though marginally all < 0.5% increased, the greatest percentage change in DALY rate from 1990 to 2019 was found in North Africa and the Middle East, followed by East Asia, Southeast Asia, and high-income North America. Given the various newly identified monogenic factors such as IL36RN and AP1S3 associated with significantly increased risk for psoriasis, perhaps the higher rate of consanguinity observed in the Middle East puts this population at greater risk for the development of psoriasis (26, 27).

Broad-scale genetic susceptibilities due to ethnicities and ancestries could also account for the difference in incidence and prevalence across various populations, particularly when considering regions of similar SDI that should have equal access to necessary diagnostic and treatment modalities (28). In fact, in the highest SDI regions, the incidence of psoriatic disease and associated YLDs was more than two times higher in central Europe than in the high-income Asia Pacific.

Our study adds to and complements the existing literature. Utilizing data from the GBD 2017, AlQassimi et al. (29) found that in 2017, the age-standardized prevalence psoriasis rate globally was 811 per 100,000 population (around 0.84% of the world population, ~64.6 million subjects). The incidence rate increased from 92 to 99 per 100,000 in 1990–2017, with the highest and the lowest rates being reported in North America and Western Europe, and in Asia and Western Pacific regions, respectively. In terms of age distribution, a peak in the incidence was noted around 55–60 years, with women being slightly more affected compared to men. Another study (30) utilized data from the GBD 2017 and came to similar conclusions. Mehrmal et al. (30) were able to find positive linear relationships between psoriasis prevalence and several comorbidities, including mental and cardiovascular disorder, stroke, metabolic impairment and diabetes, malignancies (non-Hodgkin and Hodgkin lymphoma, and non-melanoma skin cancers), and inflammatory bowel diseases, with the lowest and the highest associations being reported for stroke and non-Hodgkin lymphoma, respectively. Parisi et al. from the “Global Psoriasis Atlas” (31) deployed Bayesian inference, and the Hamiltonian Markov chain Monte Carlo method, to inform and enrich a systematic review of the literature on the global burden of psoriasis, and found that about 81% of the countries in the world lacked detailed information concerning psoriasis criteria. In adults, authors estimated an incidence rate varying from 30.3 to 321.0 per 100,000 person years in Taiwan and Italy, respectively. The prevalence of psoriasis was computed to range from 0.14% in East Asia to 1.10–1.50% in high-income southern Latin and North America, 1.83–92% in central and western Europe, and 1.99% in Australasia.

To the best of our knowledge, no study exists relying on data from the GBD 2019 study. Only two studies (32, 33) have recently reported and analyzed such data, but only partly, with one focusing only on China (32) and the other (33) investigating high-level changes in the GBD imposed by psoriasis. More in detail, utilizing an innovative decomposition-based modeling method, Xu et al. (33) have identified four major demographic and epidemiological patterns explaining the spatio-temporal heterogeneity of the global burden of psoriasis, which include (i) a substantial increase in population growth (observable in regions such as North Africa and the Middle East, Western, Eastern, and Central Sub-Saharan Africa, Andean, and Central Latin America, South Asia, and Oceania); (ii) a moderate increase in population growth (like in Western Europe, and high-income North America, Caribbean, Tropical, and Southern Latin America, Southern Sub-Saharan Africa, Southeast Asia, and Australasia); (iii) increase in population aging (observable in the high-income Asia Pacific); and (iv) combined effect of the increase in population growth and aging (as in Central, and Eastern Europe, Central, and East Asia).

There are limitations of this study, which will be briefly discussed. Psoriasis is not solely a skin disease. Approximately 30% of psoriatic patients develop psoriatic arthritis, which can be debilitating and both physically and emotionally devastating (34). Psoriatic arthritis burden is not assessed in GBD psoriasis estimates. In addition, GBD does not address the significant and potentially deadly comorbidities associated with psoriasis, including cardiovascular disease and metabolic syndrome (34). While the DW does attempt to capture worry, trouble sleeping, difficulty in concentrating, and suicidal ideation due to disfigurement and itch/pain from skin diseases, the psychological sequelae from psoriasis may be more severe than from other skin diseases and could be disproportionate to body surface area involvement (35). GBD estimation methods are dependent on the availability of data. Prevalence data sources informing psoriasis estimation represent 7 of 7 GBD super-regions, but only a part of GBD regions and countries. Geographic differences in psoriasis burden could be at least partly due to ascertainment bias in higher SDI countries. Conversely, future estimation of psoriasis burden must address the lack of data from low SDI countries, which is related to access to healthcare, diagnostic rate, and dependence on national and regional registers.

The GBD-based estimations allow for objective data to inform multiple levels of public policy. Since 2015, GBD has sought to measure progress toward sustainable development goals, which were set forth by the United National General Assembly to ensure healthy well-being for the current and future populations at large (36). Just as importantly, many countries and regions around the world have created partnerships with GBD in order to enhance local data collection systems, strengthen subnational collaborations, and ultimately, orient national healthcare strategies.

The therapeutic landscape for psoriasis has been revolutionized by biologic therapies over the most recent decade by increasing disease-free or disease-minimal periods (37). These therapies are postulated to alter the epidemiological landscape for psoriasis burden, though this has not been captured in our study. The global psoriasis treatment market is projected to generate $10.68 billion by 2022 (38). As molecular pathways of psoriasis immunopathogenesis are elucidated, a mechanistic approach to therapy has revolutionized medicine. As of the writing of this study, the list of commercially available biologic agents for psoriasis is constantly growing, including, but not limited to, inhibitors of tumor necrosis factor (TNF), interleukin (IL)-17 receptor, IL-17, IL-12/23, IL-23, phosphodiesterase, and Janus kinases. Clinical studies to assess treatment efficacy have identified tools such as the Psoriasis Area and Severity Index (PASI), with increasing benchmark endpoints of PASI 50, PASI 75, and PASI 90 (39, 40). This individual clinical approach must be balanced with a population-level view.

Concerning the strengths of the present study on a global scale, GBD 2019 assembles the most reliable epidemiological data available to estimate disease burden and, more specifically, the burden imposed by psoriasis across countries. In addition, GBD estimation is internally consistent, eliminating biases from external estimation strategies (41). The GBD process is repeated for each data iteration as new data sources are identified, and further refinements are made to analytic approaches. The fluid, high-quality, and transparent nature of GBD has transformed the epidemiological landscape for psoriasis. With future iterations, GBD strives to estimate psoriasis comorbidities including autoimmune, cardiovascular, metabolic, cutaneous, and psychiatric conditions. As more data sources become available on the local burden of disease, more precise estimates are generated at local, national, regional, super-regional, and global levels. Overall, the burden from psoriasis remains substantial, with little change over time, despite significant therapeutic advances.

## Data Availability Statement

The datasets presented in this study can be found in online repositories. The names of the repository/repositories and accession number(s) can be found in the article/Supplementary Material.

## Ethics Statement

The studies involving human participants were reviewed and approved by University of Washington Institutional Review Board (IRB). Written informed consent to participate in this study was provided by the participants' legal guardian/next of kin.

## Author Contributions

GD, CK, DW, and GA: substantial contributions to the conception or design of the work. CK, NB, GA, and RD: substantial contributions to the acquisition and substantial contributions to analysis or interpretation of data. GD, NB, and CK: drafting the work. GD, NB, CK, DW, GA, DM, CG, RD, AG, PW, CL, L-ST, KC, and MN: revising the work critically for important intellectual content and provide approval for publication of the content. All authors contributed to the article and approved the submitted version.

## Funding

GD was supported by the P50 AR070590 National Institute of Arthritis and Musculoskeletal and Skin Diseases (NIAMS) and NB was funded by the Celgene supported PARTNER fellowship program.

## Conflict of Interest

The authors declare that the research was conducted in the absence of any commercial or financial relationships that could be construed as a potential conflict of interest.

## Publisher's Note

All claims expressed in this article are solely those of the authors and do not necessarily represent those of their affiliated organizations, or those of the publisher, the editors and the reviewers. Any product that may be evaluated in this article, or claim that may be made by its manufacturer, is not guaranteed or endorsed by the publisher.
