# Supplementary material for: The Global, Regional, and National Burden of Psoriasis: Results and Insights From the Global Burden of Disease 2019 Study
Source: Front Med (Lausanne). 2021 Dec 16;8:743180. doi: 10.3389/fmed.2021.743180 (PMC8716585; doi:10.3389/fmed.2021.743180)

**SUPPLEMENTARIES**

*Supplementary Table 1*. Incidence of psoriasis in 1990 and 2019 for both sexes and percentage change of age-standardized rates, by location.

Data in parentheses are 95% uncertainty intervals. SDI= Socio-demographic Index.

| Location | 1990 |  | 2019 |  | Percentage change in age-standardised rates, 1990-2019 |
| --- | --- | --- | --- | --- | --- |
|  | Number | Age-standardized rate  (per 100 000 people) | Number | Age-standardized rate  (per 100 000 people) |  |
| Global | 3653236 (3527023 to 3778791) | 72.2 (69.7 to 74.7) | 4622594 (4458904 to 4780771) | 57.8 (55.8 to 59.7) | -20.0% (-20.2 to -19.8) |
| Low SDI | 210360 (202895 to 218246) | 45.2 (43.6 to 46.8) | 383077 (369753 to 398155) | 38.1 (36.8 to 39.5) | -15.7% (-16.1 to -15.2) |
| Low-middle SDI | 542259 (524003 to 562547) | 52.8 (50.9 to 54.6) | 778895 (751474 to 806740) | 45.1 (43.4 to 46.6) | -14.6% (-14.9 to -14.2) |
| Middle SDI | 847467 (818351 to 878420) | 53.3 (51.4 to 55.2) | 1056606 (1018549 to 1093438) | 41.7 (40.2 to 43.1) | -21.8% (-22.1 to -21.4) |
| High-middle SDI | 976580 (940735 to 1011088) | 84.2 (81.1 to 87.2) | 1106672 (1065976 to 1146076) | 69.4 (67.1 to 71.9) | -17.5% (-17.9 to -17.2) |
| High SDI | 1074706 (1038316 to 1110451) | 125.3 (121.2 to 129.4) | 1222661 (1181243 to 1262575) | 112.6 (108.9 to 116.1) | -10.2% (-10.6 to -9.7) |
| Central Sub-Saharan Africa | 30906 (29579 to 32280) | 61.8 (59.2 to 64.5) | 61984 (59320 to 64656) | 51.6 (49.4 to 53.8) | -16.5% (-17.8 to -15.0) |
| Angola | 6150 (5882 to 6453) | 65.9 (63.2 to 68.9) | 13975 (13363 to 14615) | 51.3 (49.1 to 53.5) | -22.2% (-24.0 to -20.4) |
| Central African Republic | 1642 (1572 to 1720) | 65.7 (62.9 to 68.4) | 2811 (2690 to 2945) | 57.7 (55.2 to 60.2) | -12.1% (-14.1 to -10.3) |
| Congo | 1201 (1147 to 1260) | 54.6 (52.3 to 57.2) | 2170 (2073 to 2270) | 43.4 (41.6 to 45.4) | -20.4% (-22.1 to -18.6) |
| Democratic Republic of the Congo | 21175 (20227 to 22144) | 61.1 (58.4 to 63.9) | 41824 (39992 to 43691) | 52.3 (50.0 to 54.6) | -14.4% (-16.2 to -12.4) |
| Equatorial Guinea | 255 (244 to 267) | 65.8 (62.9 to 68.7) | 520 (495 to 546) | 40.7 (38.9 to 42.6) | -38.1% (-39.6 to -36.6) |
| Gabon | 483 (461 to 505) | 53.1 (50.7 to 55.5) | 684 (654 to 715) | 40.7 (38.9 to 42.3) | -23.4% (-25.1 to -21.6) |
| Eastern Sub-Saharan Africa | 44727 (43000 to 46689) | 27.9 (26.8 to 28.9) | 89695 (86383 to 93561) | 25.1 (24.2 to 26.1) | -9.8% (-10.3 to -9.2) |
| Burundi | 1286 (1234 to 1343) | 27.3 (26.2 to 28.4) | 2716 (2610 to 2830) | 26.5 (25.5 to 27.5) | -2.9% (-4.9 to -0.7) |
| Comoros | 106 (102 to 111) | 26.7 (25.7 to 27.8) | 165 (159 to 172) | 24.4 (23.4 to 25.3) | -8.7% (-10.6 to -6.9) |
| Djibouti | 110 (105 to 114) | 26.8 (25.8 to 27.9) | 270 (259 to 281) | 24.0 (23.1 to 24.9) | -10.6% (-12.3 to -8.6) |
| Eritrea | 745 (715 to 777) | 29.3 (28.2 to 30.5) | 1546 (1485 to 1614) | 26.0 (25.0 to 27.0) | -11.4% (-13.1 to -9.5) |
| Ethiopia | 12471 (12006 to 12969) | 28.7 (27.7 to 29.7) | 23712 (22854 to 24668) | 25.6 (24.7 to 26.5) | -10.7% (-11.6 to -9.8) |
| Kenya | 4691 (4528 to 4881) | 24.4 (23.5 to 25.2) | 9999 (9644 to 10397) | 22.2 (21.4 to 23.0) | -8.9% (-9.3 to -8.6) |
| Madagascar | 2897 (2780 to 3021) | 28.4 (27.3 to 29.5) | 6225 (5988 to 6484) | 26.4 (25.4 to 27.4) | -6.9% (-8.7 to -5.0) |
| Malawi | 2363 (2271 to 2465) | 29.1 (28.0 to 30.2) | 4189 (4019 to 4357) | 26.3 (25.3 to 27.4) | -9.5% (-11.3 to -7.6) |
| Mozambique | 3594 (3451 to 3737) | 31.7 (30.5 to 32.9) | 6996 (6719 to 7291) | 28.0 (27.0 to 29.0) | -11.7% (-13.4 to -10.0) |
| Rwanda | 1470 (1380 to 1560) | 24.3 (22.9 to 25.7) | 2484 (2349 to 2629) | 21.8 (20.6 to 22.9) | -10.4% (-12.5 to -8.3) |
| Somalia | 1920 (1844 to 2002) | 31.5 (30.3 to 32.7) | 5394 (5184 to 5628) | 31.4 (30.2 to 32.6) | -0.2% (-2.2 to 1.9) |
| South Sudan | 1335 (1281 to 1400) | 26.7 (25.7 to 27.8) | 2020 (1941 to 2103) | 25.2 (24.3 to 26.2) | -5.5% (-7.3 to -3.5) |
| United Republic of Tanzania | 5932 (5573 to 6298) | 27.1 (25.5 to 28.6) | 11920 (11240 to 12660) | 24.2 (23.0 to 25.6) | -10.5% (-12.4 to -8.3) |
| Uganda | 4000 (3838 to 4179) | 27.9 (26.8 to 28.9) | 8265 (7941 to 8613) | 24.0 (23.1 to 25.0) | -13.8% (-15.4 to -12.0) |
| Zambia | 1774 (1697 to 1851) | 26.7 (25.8 to 27.8) | 3722 (3573 to 3876) | 23.7 (22.8 to 24.6) | -11.5% (-13.2 to -9.7) |
| Southern Sub-Saharan Africa | 17597 (16984 to 18313) | 36.8 (35.5 to 38.2) | 25357 (24396 to 26331) | 32.9 (31.7 to 34.1) | -10.6% (-11.3 to -10.0) |
| Botswana | 436 (419 to 455) | 38.0 (36.5 to 39.5) | 724 (694 to 757) | 31.7 (30.4 to 33.0) | -16.6% (-18.3 to -14.7) |
| Lesotho | 709 (681 to 738) | 43.5 (41.7 to 45.3) | 745 (715 to 777) | 37.2 (35.7 to 38.6) | -14.6% (-16.4 to -12.8) |
| Namibia | 470 (450 to 491) | 37.5 (36.0 to 39.1) | 728 (697 to 759) | 32.3 (31.0 to 33.6) | -14.0% (-16.1 to -12.1) |
| South Africa | 12278 (11850 to 12765) | 36.0 (34.7 to 37.2) | 17912 (17255 to 18579) | 32.1 (30.9 to 33.2) | -10.9% (-11.6 to -10.1) |
| Eswatini | 277 (265 to 291) | 40.0 (38.4 to 41.6) | 362 (347 to 378) | 34.1 (32.7 to 35.6) | -14.6% (-16.4 to -12.8) |
| Zimbabwe | 3427 (3274 to 3584) | 38.3 (36.8 to 39.9) | 4886 (4664 to 5107) | 35.8 (34.3 to 37.4) | -6.6% (-8.5 to -4.6) |
| Western Sub-Saharan Africa | 69053 (66537 to 71782) | 40.7 (39.2 to 42.1) | 131825 (127072 to 137224) | 32.5 (31.3 to 33.6) | -20.2% (-20.5 to -19.8) |
| Benin | 1515 (1455 to 1578) | 36.7 (35.4 to 38.1) | 3413 (3275 to 3550) | 31.0 (29.9 to 32.2) | -15.5% (-17.2 to -14.0) |
| Burkina Faso | 3261 (3135 to 3394) | 39.4 (37.9 to 40.9) | 6759 (6493 to 7029) | 34.0 (32.7 to 35.2) | -13.8% (-15.5 to -12.1) |
| Cameroon | 2997 (2887 to 3121) | 33.3 (32.0 to 34.5) | 7196 (6922 to 7493) | 27.8 (26.8 to 28.8) | -16.4% (-18.1 to -14.8) |
| Cabo Verde | 110 (105 to 114) | 35.6 (34.3 to 36.9) | 158 (153 to 165) | 28.5 (27.5 to 29.6) | -19.9% (-21.4 to -18.2) |
| Chad | 2112 (2031 to 2198) | 40.6 (39.2 to 42.2) | 4844 (4645 to 5043) | 34.8 (33.5 to 36.1) | -14.3% (-16.0 to -12.7) |
| Cote d'Ivoire | 3680 (3539 to 3840) | 34.9 (33.7 to 36.3) | 6979 (6708 to 7254) | 29.6 (28.5 to 30.7) | -15.3% (-17.0 to -13.5) |
| Gambia | 319 (307 to 333) | 37.5 (36.1 to 38.9) | 620 (596 to 646) | 31.1 (29.9 to 32.2) | -17.2% (-18.7 to -15.5) |
| Ghana | 5224 (4972 to 5513) | 39.5 (37.6 to 41.4) | 9664 (9184 to 10160) | 33.0 (31.4 to 34.5) | -16.5% (-18.2 to -14.8) |
| Guinea | 2069 (1987 to 2149) | 37.9 (36.5 to 39.3) | 3598 (3463 to 3747) | 32.5 (31.3 to 33.7) | -14.4% (-16.0 to -12.6) |
| Guinea-Bissau | 331 (318 to 344) | 38.0 (36.7 to 39.4) | 542 (521 to 564) | 32.1 (30.9 to 33.2) | -15.7% (-17.2 to -13.8) |
| Liberia | 619 (596 to 644) | 35.5 (34.2 to 36.9) | 1334 (1284 to 1386) | 30.6 (29.5 to 31.7) | -13.9% (-15.5 to -12.2) |
| Mali | 2625 (2481 to 2771) | 34.8 (33.0 to 36.5) | 5376 (5061 to 5695) | 28.7 (27.2 to 30.2) | -17.5% (-19.3 to -15.8) |
| Mauritania | 648 (623 to 674) | 35.8 (34.5 to 37.2) | 1089 (1047 to 1133) | 30.0 (28.8 to 31.1) | -16.3% (-17.9 to -14.5) |
| Niger | 2860 (2744 to 2975) | 41.7 (40.1 to 43.2) | 7343 (7053 to 7658) | 37.2 (35.8 to 38.6) | -10.7% (-12.4 to -9.1) |
| Nigeria | 35937 (34687 to 37281) | 44.4 (42.9 to 45.9) | 64049 (61790 to 66559) | 33.4 (32.2 to 34.5) | -24.8% (-25.3 to -24.4) |
| Sao Tome and Principe | 35 (33 to 36) | 32.8 (31.6 to 33.9) | 51 (49 to 53) | 26.9 (25.9 to 27.9) | -17.9% (-19.5 to -16.3) |
| Senegal | 2430 (2332 to 2531) | 37.0 (35.6 to 38.4) | 4312 (4149 to 4483) | 31.6 (30.4 to 32.8) | -14.7% (-16.4 to -12.9) |
| Sierra Leone | 1194 (1150 to 1239) | 36.8 (35.5 to 38.2) | 2344 (2249 to 2439) | 31.6 (30.5 to 32.8) | -14.0% (-15.8 to -12.3) |
| Togo | 1086 (1043 to 1134) | 34.9 (33.6 to 36.2) | 2152 (2071 to 2241) | 29.8 (28.7 to 30.9) | -14.7% (-16.3 to -12.9) |
| Andean Latin America | 33750 (32270 to 35227) | 94.8 (90.9 to 98.8) | 52103 (49796 to 54275) | 82.4 (78.8 to 85.8) | -13.1% (-14.3 to -11.9) |
| Bolivia (Plurinational State of) | 6392 (6131 to 6672) | 107.3 (102.8 to 111.8) | 10806 (10336 to 11272) | 92.2 (88.1 to 96.1) | -14.1% (-16.0 to -12.2) |
| Ecuador | 8308 (7944 to 8692) | 89.0 (85.1 to 93.1) | 13354 (12779 to 13967) | 76.7 (73.5 to 80.2) | -13.8% (-15.7 to -12.0) |
| Peru | 19050 (18164 to 19900) | 93.8 (89.8 to 98.0) | 27942 (26655 to 29131) | 81.9 (78.2 to 85.2) | -12.8% (-14.6 to -11.0) |
| Tropical Latin America | 134553 (129923 to 139258) | 92.4 (89.2 to 95.4) | 202572 (195312 to 209349) | 87.1 (84.1 to 90.0) | -5.7% (-6.2 to -5.1) |
| Brazil | 130999 (126494 to 135573) | 92.3 (89.1 to 95.3) | 196399 (189359 to 202875) | 87.0 (84.0 to 89.9) | -5.7% (-6.3 to -5.1) |
| Paraguay | 3554 (3397 to 3711) | 94.8 (90.7 to 98.9) | 6172 (5902 to 6455) | 90.1 (86.2 to 94.1) | -4.9% (-6.9 to -2.8) |
| Central Latin America | 34843 (33618 to 36214) | 23.6 (22.7 to 24.5) | 52370 (50378 to 54362) | 20.7 (19.9 to 21.5) | -12.2% (-12.7 to -11.7) |
| Colombia | 6574 (6312 to 6837) | 22.0 (21.2 to 22.8) | 9471 (9106 to 9861) | 19.2 (18.4 to 19.9) | -12.9% (-14.6 to -11.2) |
| Costa Rica | 602 (578 to 626) | 21.7 (20.8 to 22.5) | 938 (899 to 974) | 19.0 (18.3 to 19.7) | -12.2% (-13.8 to -10.6) |
| El Salvador | 1174 (1130 to 1222) | 25.0 (24.0 to 25.9) | 1313 (1260 to 1369) | 21.3 (20.5 to 22.2) | -14.6% (-16.3 to -13.0) |
| Guatemala | 1897 (1821 to 1978) | 27.2 (26.2 to 28.3) | 3742 (3588 to 3906) | 22.6 (21.7 to 23.5) | -17.0% (-18.7 to -15.4) |
| Honduras | 1088 (1042 to 1136) | 26.8 (25.7 to 27.9) | 2128 (2050 to 2223) | 23.2 (22.3 to 24.1) | -13.4% (-15.3 to -11.5) |
| Mexico | 18444 (17794 to 19178) | 24.1 (23.2 to 24.9) | 26783 (25781 to 27818) | 21.1 (20.3 to 21.9) | -12.4% (-12.8 to -11.9) |
| Nicaragua | 864 (831 to 902) | 26.0 (25.0 to 27.0) | 1397 (1341 to 1454) | 22.2 (21.4 to 23.1) | -14.7% (-16.4 to -12.9) |
| Panama | 469 (451 to 488) | 21.2 (20.4 to 22.0) | 786 (755 to 815) | 18.8 (18.1 to 19.5) | -11.4% (-13.1 to -9.6) |
| Venezuela (Bolivarian Republic of) | 3731 (3581 to 3878) | 21.9 (21.0 to 22.7) | 5812 (5578 to 6044) | 19.9 (19.2 to 20.7) | -8.9% (-10.6 to -7.1) |
| Southern Latin America | 54019 (51757 to 56252) | 110.1 (105.5 to 114.7) | 70211 (67264 to 73298) | 102.1 (97.9 to 106.3) | -7.3% (-8.8 to -5.7) |
| Argentina | 36166 (34666 to 37684) | 110.1 (105.4 to 114.9) | 47387 (45347 to 49524) | 102.9 (98.5 to 107.3) | -6.5% (-8.7 to -4.4) |
| Chile | 14419 (13782 to 15057) | 110.6 (105.8 to 115.4) | 19225 (18381 to 20058) | 100.2 (95.9 to 104.2) | -9.5% (-11.5 to -7.4) |
| Uruguay | 3432 (3291 to 3578) | 108.4 (103.9 to 113.2) | 3596 (3445 to 3758) | 101.8 (97.5 to 106.3) | -6.1% (-8.2 to -4.0) |
| Caribbean | 20607 (19829 to 21437) | 61.3 (58.9 to 63.7) | 27467 (26394 to 28614) | 56.9 (54.7 to 59.2) | -7.1% (-8.1 to -6.1) |
| Antigua and Barbuda | 32 (31 to 34) | 55.6 (53.3 to 58.0) | 48 (46 to 50) | 50.3 (48.2 to 52.4) | -9.6% (-11.5 to -7.6) |
| Bahamas | 131 (126 to 137) | 53.8 (51.7 to 56.1) | 200 (191 to 208) | 50.0 (47.9 to 52.0) | -7.1% (-9.3 to -5.0) |
| Barbados | 132 (127 to 137) | 52.6 (50.5 to 54.8) | 161 (154 to 168) | 49.1 (47.1 to 51.2) | -6.8% (-8.7 to -4.5) |
| Belize | 109 (104 to 113) | 65.9 (63.2 to 68.4) | 225 (216 to 235) | 56.5 (54.2 to 58.8) | -14.3% (-16.2 to -12.5) |
| Bermuda | 35 (34 to 37) | 56.5 (54.3 to 58.9) | 37 (36 to 39) | 51.2 (49.1 to 53.3) | -9.5% (-11.5 to -7.5) |
| Cuba | 6293 (6037 to 6577) | 58.3 (56.0 to 60.8) | 6896 (6572 to 7209) | 54.7 (52.4 to 57.0) | -6.3% (-8.2 to -4.4) |
| Dominica | 40 (38 to 41) | 56.9 (54.7 to 59.2) | 37 (35 to 38) | 50.5 (48.6 to 52.6) | -11.2% (-13.2 to -9.3) |
| Dominican Republic | 4275 (4088 to 4467) | 65.1 (62.3 to 67.9) | 6164 (5902 to 6431) | 57.0 (54.6 to 59.4) | -12.4% (-14.2 to -10.5) |
| Grenada | 48 (46 to 50) | 61.1 (58.6 to 63.7) | 57 (54 to 59) | 52.3 (50.1 to 54.5) | -14.4% (-16.2 to -12.4) |
| Guyana | 410 (392 to 429) | 58.2 (55.8 to 60.5) | 401 (383 to 418) | 52.0 (49.9 to 54.1) | -10.6% (-12.7 to -8.6) |
| Haiti | 4175 (4005 to 4349) | 71.9 (68.9 to 74.8) | 7689 (7367 to 8012) | 65.2 (62.6 to 67.8) | -9.3% (-11.2 to -7.4) |
| Jamaica | 1307 (1253 to 1362) | 59.7 (57.2 to 62.2) | 1523 (1457 to 1592) | 52.9 (50.8 to 55.2) | -11.4% (-13.3 to -9.6) |
| Puerto Rico | 1904 (1824 to 1984) | 52.9 (50.7 to 55.1) | 1830 (1751 to 1908) | 47.6 (45.7 to 49.5) | -10.0% (-12.1 to -8.1) |
| Saint Kitts and Nevis | 22 (21 to 23) | 56.3 (54.0 to 58.7) | 32 (31 to 34) | 49.9 (47.9 to 51.9) | -11.5% (-13.3 to -9.7) |
| Saint Lucia | 75 (72 to 78) | 59.4 (57.1 to 62.0) | 99 (94 to 103) | 52.4 (50.2 to 54.6) | -11.8% (-13.6 to -10.0) |
| Saint Vincent and the Grenadines | 61 (59 to 64) | 61.1 (58.5 to 63.5) | 64 (61 to 67) | 53.9 (51.5 to 56.1) | -11.8% (-13.7 to -9.9) |
| Suriname | 206 (198 to 216) | 56.4 (54.2 to 58.7) | 303 (291 to 316) | 50.8 (48.8 to 52.9) | -9.9% (-11.7 to -8.0) |
| Trinidad and Tobago | 609 (584 to 636) | 53.1 (51.0 to 55.3) | 718 (687 to 749) | 47.6 (45.7 to 49.6) | -10.2% (-12.1 to -8.3) |
| United States Virgin Islands | 57 (55 to 59) | 53.5 (51.4 to 55.8) | 54 (51 to 56) | 46.9 (45.0 to 48.9) | -12.4% (-14.3 to -10.5) |
| Central Europe | 92543 (89254 to 95815) | 71.3 (68.8 to 73.8) | 76996 (74396 to 79469) | 60.3 (58.5 to 62.2) | -15.5% (-16.3 to -14.6) |
| Albania | 2358 (2265 to 2450) | 74.9 (72.1 to 77.7) | 1814 (1743 to 1884) | 60.9 (58.7 to 63.2) | -18.7% (-20.5 to -17.0) |
| Bosnia and Herzegovina | 3610 (3473 to 3749) | 76.3 (73.6 to 79.2) | 2269 (2174 to 2358) | 60.1 (57.9 to 62.4) | -21.2% (-22.9 to -19.4) |
| Bulgaria | 6684 (6419 to 6938) | 70.4 (67.8 to 73.1) | 4552 (4366 to 4734) | 58.1 (55.8 to 60.3) | -17.5% (-19.3 to -15.6) |
| Croatia | 3674 (3527 to 3824) | 69.0 (66.5 to 71.7) | 2744 (2635 to 2853) | 57.5 (55.3 to 59.5) | -16.7% (-18.4 to -14.8) |
| Czechia | 7673 (7378 to 7957) | 69.9 (67.2 to 72.5) | 6795 (6517 to 7069) | 57.2 (55.1 to 59.2) | -18.1% (-19.9 to -16.3) |
| Hungary | 7939 (7616 to 8224) | 70.7 (67.9 to 73.2) | 6317 (6055 to 6548) | 58.1 (56.0 to 60.3) | -17.9% (-19.7 to -16.2) |
| North Macedonia | 1468 (1413 to 1519) | 71.0 (68.4 to 73.6) | 1422 (1363 to 1476) | 58.6 (56.4 to 60.7) | -17.5% (-19.2 to -15.7) |
| Montenegro | 433 (416 to 448) | 67.2 (64.7 to 69.6) | 392 (377 to 408) | 56.9 (54.7 to 58.9) | -15.5% (-17.3 to -13.7) |
| Poland | 29206 (28137 to 30311) | 73.5 (70.7 to 76.2) | 28151 (27328 to 28936) | 65.8 (64.1 to 67.4) | -10.4% (-12.4 to -8.5) |
| Romania | 16954 (16323 to 17555) | 68.5 (66.1 to 71.1) | 12055 (11615 to 12533) | 55.8 (53.8 to 57.8) | -18.6% (-20.3 to -16.8) |
| Serbia | 7233 (6955 to 7503) | 71.3 (68.7 to 74.0) | 5654 (5437 to 5877) | 58.3 (56.2 to 60.4) | -18.2% (-20.1 to -16.3) |
| Slovakia | 3900 (3761 to 4042) | 71.4 (68.8 to 74.0) | 3526 (3388 to 3661) | 57.5 (55.4 to 59.7) | -19.5% (-21.3 to -17.8) |
| Slovenia | 1412 (1358 to 1466) | 67.2 (64.6 to 69.6) | 1305 (1252 to 1356) | 55.9 (53.8 to 57.9) | -16.8% (-18.7 to -15.1) |
| Eastern Europe | 161524 (155673 to 167380) | 67.1 (64.8 to 69.6) | 139976 (134745 to 144910) | 59.5 (57.6 to 61.5) | -11.3% (-11.9 to -10.6) |
| Belarus | 8076 (7754 to 8369) | 73.0 (70.3 to 75.7) | 6510 (6251 to 6781) | 60.9 (58.6 to 63.2) | -16.7% (-18.4 to -14.7) |
| Estonia | 1175 (1129 to 1220) | 70.6 (67.9 to 73.3) | 848 (814 to 879) | 58.8 (56.6 to 60.9) | -16.7% (-18.5 to -14.7) |
| Latvia | 1975 (1900 to 2052) | 69.7 (67.2 to 72.3) | 1246 (1195 to 1297) | 58.7 (56.5 to 60.9) | -15.8% (-17.5 to -13.9) |
| Lithuania | 2680 (2574 to 2778) | 69.5 (66.8 to 72.1) | 1791 (1717 to 1863) | 57.6 (55.4 to 59.7) | -17.2% (-18.9 to -15.2) |
| Republic of Moldova | 3244 (3125 to 3362) | 71.2 (68.6 to 73.9) | 2553 (2448 to 2653) | 61.3 (58.9 to 63.5) | -14.0% (-15.8 to -12.0) |
| Russian Federation | 105532 (101563 to 109520) | 65.9 (63.5 to 68.4) | 96948 (93468 to 100400) | 59.2 (57.3 to 61.2) | -10.2% (-10.9 to -9.4) |
| Ukraine | 38843 (37424 to 40324) | 68.6 (66.1 to 71.1) | 30080 (28899 to 31319) | 60.4 (58.1 to 62.6) | -12.0% (-13.9 to -10.2) |
| North Africa and Middle East | 224312 (215902 to 233060) | 72.1 (69.4 to 74.9) | 336787 (324072 to 349591) | 55.5 (53.5 to 57.5) | -23.1% (-23.7 to -22.5) |
| Afghanistan | 8719 (8373 to 9083) | 85.8 (82.4 to 89.3) | 23306 (22374 to 24298) | 70.9 (68.3 to 73.8) | -17.3% (-19.0 to -15.6) |
| Algeria | 15261 (14634 to 15902) | 67.9 (65.1 to 70.7) | 23015 (22050 to 23987) | 54.2 (52.1 to 56.4) | -20.2% (-22.1 to -18.6) |
| Bahrain | 298 (284 to 312) | 60.8 (58.3 to 63.3) | 827 (788 to 865) | 49.9 (48.0 to 51.9) | -17.9% (-19.8 to -15.9) |
| Egypt | 21844 (20908 to 22743) | 43.4 (41.5 to 45.1) | 32864 (31645 to 34097) | 34.5 (33.3 to 35.8) | -20.4% (-22.1 to -18.6) |
| Iran (Islamic Republic of) | 37217 (35895 to 38552) | 71.6 (69.2 to 74.0) | 48309 (46547 to 50098) | 54.4 (52.5 to 56.3) | -24.0% (-24.5 to -23.5) |
| Iraq | 12168 (11639 to 12696) | 79.3 (75.9 to 82.7) | 24332 (23393 to 25350) | 60.8 (58.5 to 63.3) | -23.3% (-25.0 to -21.7) |
| Jordan | 2118 (2023 to 2212) | 64.9 (62.0 to 67.5) | 5801 (5570 to 6036) | 51.7 (49.7 to 53.7) | -20.2% (-21.9 to -18.4) |
| Kuwait | 952 (909 to 999) | 56.1 (53.7 to 58.6) | 2264 (2169 to 2365) | 46.6 (44.8 to 48.4) | -16.9% (-18.8 to -15.0) |
| Lebanon | 2082 (1998 to 2168) | 67.9 (65.2 to 70.7) | 2785 (2678 to 2900) | 52.7 (50.6 to 54.8) | -22.4% (-23.9 to -20.6) |
| Libya | 2400 (2303 to 2500) | 64.8 (62.1 to 67.5) | 3566 (3415 to 3723) | 50.3 (48.3 to 52.4) | -22.4% (-24.0 to -20.7) |
| Morocco | 17474 (16774 to 18249) | 75.2 (72.2 to 78.3) | 22227 (21324 to 23124) | 60.2 (57.8 to 62.6) | -20.0% (-21.7 to -18.2) |
| Palestine | 1355 (1301 to 1414) | 76.8 (73.5 to 80.0) | 2587 (2487 to 2694) | 57.0 (54.7 to 59.2) | -25.8% (-27.4 to -24.1) |
| Oman | 1178 (1128 to 1230) | 67.0 (64.3 to 69.7) | 2234 (2120 to 2343) | 46.9 (45.1 to 48.8) | -29.9% (-31.6 to -28.3) |
| Qatar | 263 (250 to 276) | 58.7 (56.3 to 61.1) | 1458 (1383 to 1536) | 45.8 (44.0 to 47.6) | -22.0% (-23.9 to -20.0) |
| Saudi Arabia | 10252 (9807 to 10729) | 71.1 (68.0 to 74.3) | 18070 (17237 to 18853) | 47.3 (45.3 to 49.1) | -33.5% (-35.1 to -31.9) |
| Sudan | 13214 (12688 to 13747) | 74.9 (71.8 to 78.1) | 20787 (19974 to 21670) | 56.6 (54.4 to 58.9) | -24.4% (-26.1 to -22.7) |
| Syrian Arab Republic | 8111 (7754 to 8447) | 73.0 (70.1 to 75.9) | 8321 (7973 to 8668) | 57.3 (55.0 to 59.6) | -21.6% (-23.2 to -19.9) |
| Tunisia | 5403 (5168 to 5628) | 69.3 (66.3 to 72.1) | 6757 (6485 to 7022) | 54.8 (52.7 to 57.0) | -20.9% (-22.5 to -19.1) |
| Turkey | 53717 (51480 to 56046) | 95.4 (91.5 to 99.3) | 65172 (62562 to 67837) | 74.7 (71.9 to 77.5) | -21.7% (-23.3 to -19.9) |
| United Arab Emirates | 1046 (997 to 1101) | 57.1 (54.8 to 59.5) | 4793 (4543 to 5056) | 44.0 (42.2 to 45.7) | -23.0% (-24.9 to -21.1) |
| Yemen | 9088 (8728 to 9493) | 78.3 (75.3 to 81.4) | 16968 (16269 to 17688) | 60.5 (58.1 to 63.0) | -22.7% (-24.2 to -21.1) |
| Central Asia | 48180 (46156 to 50094) | 74.1 (71.2 to 77.1) | 58976 (56469 to 61360) | 62.2 (59.7 to 64.7) | -16.0% (-16.9 to -15.0) |
| Armenia | 2462 (2351 to 2567) | 72.9 (69.8 to 76.0) | 1941 (1851 to 2031) | 59.1 (56.5 to 61.6) | -19.0% (-20.8 to -17.3) |
| Azerbaijan | 4896 (4675 to 5113) | 70.1 (67.2 to 73.0) | 6606 (6297 to 6907) | 59.7 (57.2 to 62.3) | -14.8% (-16.7 to -12.9) |
| Georgia | 3886 (3712 to 4056) | 67.7 (64.7 to 70.6) | 2377 (2268 to 2484) | 59.7 (57.0 to 62.4) | -11.8% (-13.7 to -9.7) |
| Kazakhstan | 11705 (11192 to 12199) | 73.1 (70.0 to 76.3) | 11534 (11012 to 12029) | 60.8 (58.2 to 63.5) | -16.8% (-18.6 to -14.8) |
| Kyrgyzstan | 3103 (2965 to 3243) | 75.1 (71.8 to 78.3) | 4183 (3997 to 4370) | 65.5 (62.7 to 68.4) | -12.7% (-14.7 to -10.8) |
| Mongolia | 1514 (1449 to 1581) | 79.1 (75.7 to 82.5) | 2210 (2113 to 2302) | 64.8 (62.1 to 67.4) | -18.1% (-19.8 to -16.2) |
| Tajikistan | 3674 (3515 to 3838) | 77.9 (74.5 to 81.3) | 6039 (5788 to 6298) | 66.7 (64.0 to 69.4) | -14.3% (-16.2 to -12.5) |
| Turkmenistan | 2412 (2309 to 2517) | 72.6 (69.6 to 75.7) | 2979 (2851 to 3115) | 58.5 (56.0 to 61.1) | -19.4% (-21.3 to -17.5) |
| Uzbekistan | 14527 (13902 to 15160) | 77.5 (74.2 to 80.7) | 21107 (20180 to 22041) | 62.9 (60.2 to 65.5) | -18.7% (-20.6 to -16.8) |
| South Asia | 491106 (474325 to 509187) | 48.4 (46.8 to 50.1) | 792848 (765638 to 821309) | 44.4 (42.9 to 46.0) | -8.2% (-8.6 to -7.8) |
| Bangladesh | 45289 (43561 to 47173) | 46.8 (45.2 to 48.6) | 62114 (59617 to 64412) | 39.2 (37.7 to 40.7) | -16.3% (-18.0 to -14.6) |
| Bhutan | 266 (256 to 277) | 48.7 (47.0 to 50.5) | 308 (297 to 321) | 41.1 (39.6 to 42.6) | -15.7% (-17.2 to -14.1) |
| India | 388949 (375495 to 403587) | 48.6 (46.9 to 50.3) | 635346 (613080 to 657984) | 45.7 (44.1 to 47.3) | -6.0% (-6.4 to -5.5) |
| Nepal | 7975 (7691 to 8282) | 45.3 (43.7 to 47.0) | 10310 (9936 to 10714) | 35.0 (33.8 to 36.3) | -22.8% (-24.5 to -21.2) |
| Pakistan | 48627 (46956 to 50473) | 48.5 (46.9 to 50.3) | 84769 (81758 to 88026) | 41.1 (39.6 to 42.6) | -15.3% (-16.4 to -14.3) |
| Southeast Asia | 94621 (91120 to 98283) | 23.0 (22.2 to 23.9) | 139036 (133404 to 144184) | 20.1 (19.3 to 20.8) | -12.9% (-13.4 to -12.4) |
| Cambodia | 2655 (2546 to 2768) | 31.3 (30.1 to 32.5) | 4215 (4048 to 4395) | 26.6 (25.6 to 27.7) | -15.0% (-16.7 to -13.2) |
| Indonesia | 23160 (22248 to 24050) | 14.3 (13.8 to 14.8) | 34475 (33118 to 35772) | 12.9 (12.4 to 13.4) | -9.8% (-10.4 to -9.2) |
| Lao People's Democratic Republic | 1127 (1082 to 1173) | 32.2 (30.9 to 33.5) | 1812 (1740 to 1885) | 27.2 (26.2 to 28.2) | -15.6% (-17.4 to -13.8) |
| Malaysia | 3625 (3477 to 3769) | 23.4 (22.5 to 24.2) | 6234 (5979 to 6493) | 19.7 (18.9 to 20.5) | -15.6% (-17.4 to -14.0) |
| Maldives | 51 (49 to 53) | 28.4 (27.3 to 29.5) | 115 (110 to 121) | 22.9 (22.0 to 23.8) | -19.2% (-20.8 to -17.5) |
| Mauritius | 272 (261 to 282) | 26.2 (25.2 to 27.2) | 331 (316 to 345) | 22.6 (21.7 to 23.5) | -13.6% (-15.4 to -11.8) |
| Myanmar | 11465 (11016 to 11948) | 31.7 (30.6 to 33.0) | 14468 (13896 to 15026) | 26.5 (25.4 to 27.4) | -16.7% (-18.4 to -14.9) |
| Philippines | 14330 (13826 to 14865) | 26.6 (25.7 to 27.5) | 25748 (24810 to 26703) | 24.3 (23.4 to 25.2) | -8.6% (-9.0 to -8.3) |
| Sri Lanka | 6841 (6555 to 7153) | 42.5 (40.8 to 44.4) | 8196 (7838 to 8565) | 34.9 (33.4 to 36.4) | -18.0% (-19.7 to -16.4) |
| Seychelles | 16 (15 to 16) | 23.4 (22.5 to 24.3) | 23 (22 to 24) | 20.7 (19.8 to 21.5) | -11.5% (-13.3 to -9.7) |
| Thailand | 13679 (13113 to 14293) | 25.7 (24.6 to 26.7) | 18373 (17574 to 19165) | 22.3 (21.4 to 23.2) | -13.1% (-14.9 to -11.3) |
| Timor-Leste | 199 (191 to 207) | 30.7 (29.5 to 31.9) | 297 (285 to 309) | 25.5 (24.5 to 26.5) | -17.0% (-18.8 to -15.4) |
| Viet Nam | 17077 (16389 to 17824) | 29.0 (27.8 to 30.2) | 24566 (23509 to 25523) | 24.0 (23.0 to 24.9) | -17.1% (-18.8 to -15.4) |
| East Asia | 813617 (784061 to 843006) | 68.4 (65.9 to 70.8) | 925967 (891325 to 958216) | 54.1 (52.2 to 55.9) | -20.8% (-21.3 to -20.4) |
| China | 784605 (756135 to 813047) | 68.2 (65.8 to 70.7) | 892906 (859645 to 924276) | 54.0 (52.1 to 55.8) | -20.9% (-21.4 to -20.5) |
| Democratic People's Republic of Korea | 16106 (15424 to 16801) | 78.6 (75.5 to 81.9) | 18993 (18187 to 19767) | 65.6 (63.0 to 68.2) | -16.5% (-18.4 to -14.8) |
| Taiwan (Province of China) | 12906 (12374 to 13462) | 63.6 (61.1 to 66.3) | 14068 (13426 to 14653) | 50.2 (48.2 to 52.0) | -21.1% (-23.0 to -19.3) |
| Oceania | 2382 (2286 to 2479) | 43.1 (41.4 to 44.9) | 4694 (4512 to 4889) | 39.2 (37.6 to 40.8) | -9.1% (-10.5 to -7.7) |
| American Samoa | 15 (14 to 16) | 36.0 (34.5 to 37.5) | 17 (17 to 18) | 31.9 (30.6 to 33.1) | -11.4% (-13.3 to -9.6) |
| Federated States of Micronesia | 34 (32 to 35) | 39.5 (37.9 to 41.1) | 34 (32 to 35) | 34.5 (33.1 to 35.9) | -12.6% (-14.5 to -10.5) |
| Cook Islands | 6 (6 to 6) | 35.7 (34.2 to 37.2) | 6 (6 to 6) | 31.1 (29.8 to 32.3) | -13.0% (-14.9 to -11.0) |
| Nauru | 3 (3 to 3) | 35.2 (33.7 to 36.6) | 3 (3 to 3) | 31.4 (30.2 to 32.7) | -10.7% (-12.6 to -8.7) |
| Niue | 1 (1 to 1) | 37.3 (35.8 to 38.9) | 1 (1 to 1) | 32.4 (31.1 to 33.7) | -13.2% (-15.0 to -11.3) |
| Palau | 5 (5 to 5) | 33.9 (32.5 to 35.4) | 6 (6 to 7) | 30.3 (29.0 to 31.5) | -10.7% (-12.6 to -8.7) |
| Fiji | 263 (252 to 276) | 39.2 (37.6 to 40.9) | 312 (299 to 325) | 34.2 (32.8 to 35.6) | -12.6% (-14.4 to -10.8) |
| Guam | 42 (40 to 44) | 33.0 (31.6 to 34.3) | 51 (49 to 54) | 29.1 (27.9 to 30.3) | -11.6% (-13.5 to -9.6) |
| Kiribati | 25 (24 to 26) | 39.2 (37.7 to 40.7) | 40 (38 to 42) | 37.0 (35.5 to 38.5) | -5.6% (-7.6 to -3.7) |
| Marshall Islands | 15 (14 to 16) | 41.9 (40.1 to 43.6) | 19 (18 to 20) | 35.7 (34.3 to 37.2) | -14.6% (-16.5 to -12.8) |
| Northern Mariana Islands | 14 (14 to 15) | 33.6 (32.2 to 35.1) | 15 (15 to 16) | 31.1 (29.9 to 32.4) | -7.3% (-9.5 to -5.2) |
| Papua New Guinea | 1555 (1490 to 1622) | 44.8 (42.9 to 46.6) | 3524 (3382 to 3678) | 40.3 (38.7 to 41.9) | -10.1% (-11.9 to -8.2) |
| Samoa | 53 (51 to 55) | 38.2 (36.7 to 39.8) | 70 (68 to 73) | 36.2 (34.8 to 37.7) | -5.2% (-7.3 to -3.0) |
| Solomon Islands | 126 (121 to 132) | 45.8 (43.9 to 47.6) | 231 (222 to 241) | 40.3 (38.7 to 42.0) | -11.9% (-13.6 to -10.0) |
| Tokelau | 1 (1 to 1) | 38.3 (36.7 to 39.8) | 0 (0 to 0) | 32.5 (31.2 to 33.8) | -15.0% (-16.9 to -13.3) |
| Tonga | 33 (32 to 35) | 40.3 (38.6 to 42.0) | 34 (32 to 35) | 35.7 (34.1 to 37.1) | -11.5% (-13.3 to -9.6) |
| Tuvalu | 3 (3 to 4) | 39.2 (37.6 to 40.7) | 4 (4 to 4) | 34.1 (32.6 to 35.4) | -13.0% (-14.9 to -11.0) |
| Vanuatu | 55 (53 to 58) | 44.3 (42.4 to 46.1) | 103 (98 to 108) | 39.2 (37.6 to 40.8) | -11.4% (-13.3 to -9.5) |
| High-income Asia Pacific | 76336 (73621 to 79135) | 41.4 (39.9 to 42.9) | 83991 (80923 to 86862) | 40.0 (38.5 to 41.4) | -3.4% (-4.0 to -2.7) |
| Brunei Darussalam | 89 (86 to 93) | 37.4 (36.0 to 38.8) | 161 (154 to 168) | 34.9 (33.5 to 36.3) | -6.7% (-8.5 to -4.8) |
| Japan | 55544 (53578 to 57514) | 40.5 (39.1 to 41.9) | 57045 (54998 to 58992) | 40.4 (39.0 to 41.8) | -0.3% (-0.7 to 0.1) |
| Singapore | 1126 (1080 to 1174) | 36.0 (34.6 to 37.4) | 2164 (2074 to 2250) | 33.0 (31.8 to 34.3) | -8.2% (-10.1 to -6.2) |
| Republic of Korea | 19576 (18790 to 20437) | 44.2 (42.5 to 45.9) | 24621 (23555 to 25646) | 39.9 (38.3 to 41.5) | -9.7% (-11.6 to -7.7) |
| High-income North America | 302143 (292313 to 311286) | 105.0 (101.7 to 108.1) | 359271 (347584 to 370502) | 92.7 (89.7 to 95.5) | -11.8% (-12.5 to -11.0) |
| Canada | 27254 (26311 to 28143) | 96.3 (93.1 to 99.4) | 34709 (33461 to 35993) | 88.5 (85.7 to 91.7) | -8.1% (-9.8 to -6.3) |
| Greenland | 66 (64 to 69) | 116.6 (113.0 to 120.5) | 64 (61 to 66) | 104.6 (101.1 to 108.1) | -10.3% (-12.0 to -8.6) |
| United States of America | 274815 (266032 to 283181) | 106.0 (102.7 to 109.1) | 324492 (313901 to 334530) | 93.2 (90.3 to 96.0) | -12.1% (-12.9 to -11.3) |
| Western Europe | 873918 (842795 to 904003) | 216.9 (209.3 to 224.2) | 946916 (913064 to 979530) | 204.5 (197.6 to 211.4) | -5.7% (-6.2 to -5.2) |
| Andorra | 124 (119 to 128) | 212.9 (205.5 to 220.2) | 193 (186 to 201) | 205.1 (198.0 to 212.5) | -3.7% (-5.6 to -1.7) |
| Austria | 18745 (18076 to 19420) | 229.7 (221.2 to 237.7) | 20770 (19961 to 21523) | 216.2 (208.5 to 223.8) | -5.9% (-7.6 to -4.0) |
| Belgium | 24611 (23699 to 25517) | 234.1 (225.6 to 242.2) | 26452 (25453 to 27481) | 218.9 (211.0 to 227.0) | -6.5% (-8.4 to -4.6) |
| Cyprus | 1815 (1749 to 1877) | 229.1 (220.9 to 237.0) | 2924 (2811 to 3032) | 204.9 (197.3 to 212.1) | -10.6% (-12.4 to -8.5) |
| Denmark | 12448 (11992 to 12905) | 230.4 (222.2 to 238.5) | 13386 (12870 to 13875) | 218.2 (210.6 to 225.9) | -5.3% (-7.1 to -3.5) |
| Finland | 13102 (12649 to 13559) | 247.7 (239.4 to 256.3) | 13501 (13010 to 13980) | 232.8 (224.4 to 241.0) | -6.0% (-7.8 to -4.2) |
| France | 160279 (154148 to 166526) | 269.4 (259.3 to 279.8) | 172843 (166210 to 179240) | 251.7 (242.5 to 261.0) | -6.6% (-8.4 to -4.6) |
| Germany | 162807 (156861 to 168814) | 188.7 (181.9 to 195.7) | 167515 (161352 to 173618) | 182.2 (176.0 to 188.5) | -3.5% (-5.4 to -1.4) |
| Greece | 25418 (24479 to 26360) | 231.8 (223.7 to 240.3) | 23753 (22836 to 24627) | 215.6 (208.2 to 223.1) | -7.0% (-8.8 to -5.1) |
| Iceland | 630 (607 to 652) | 246.9 (238.4 to 255.2) | 833 (802 to 862) | 230.0 (222.0 to 238.3) | -6.8% (-8.8 to -4.9) |
| Ireland | 8591 (8295 to 8880) | 239.5 (231.2 to 247.8) | 11346 (10944 to 11771) | 218.9 (211.5 to 226.9) | -8.6% (-10.6 to -6.8) |
| Israel | 10556 (10194 to 10927) | 218.5 (211.2 to 225.9) | 19158 (18527 to 19854) | 206.3 (199.5 to 214.1) | -5.6% (-7.6 to -3.6) |
| Italy | 110243 (106453 to 113988) | 184.4 (178.3 to 190.4) | 109807 (105647 to 114073) | 172.8 (167.0 to 178.7) | -6.3% (-6.9 to -5.7) |
| Luxembourg | 902 (870 to 934) | 221.3 (213.9 to 228.9) | 1427 (1374 to 1476) | 212.1 (204.7 to 219.3) | -4.2% (-6.1 to -2.2) |
| Malta | 891 (860 to 924) | 231.2 (223.2 to 239.6) | 996 (958 to 1032) | 211.6 (204.0 to 219.0) | -8.5% (-10.4 to -6.6) |
| Monaco | 70 (67 to 72) | 212.9 (205.9 to 220.3) | 83 (80 to 86) | 204.5 (197.3 to 211.7) | -4.0% (-5.8 to -2.1) |
| Netherlands | 35699 (34429 to 36965) | 228.0 (220.0 to 235.9) | 39506 (37991 to 41047) | 215.6 (207.8 to 223.4) | -5.4% (-7.3 to -3.6) |
| Norway | 7564 (7318 to 7813) | 173.4 (167.9 to 179.1) | 9355 (9038 to 9670) | 165.3 (160.0 to 170.7) | -4.7% (-5.2 to -4.2) |
| Portugal | 25800 (24865 to 26716) | 244.6 (235.6 to 253.1) | 25564 (24604 to 26526) | 223.7 (215.5 to 231.8) | -8.6% (-10.3 to -6.7) |
| San Marino | 54 (52 to 55) | 217.5 (210.1 to 225.2) | 73 (70 to 76) | 207.7 (200.2 to 215.1) | -4.5% (-6.3 to -2.6) |
| Spain | 90544 (86844 to 94095) | 224.6 (215.3 to 233.6) | 104133 (99854 to 108476) | 209.0 (200.7 to 217.3) | -7.0% (-8.8 to -5.2) |
| Sweden | 16171 (15582 to 16725) | 179.4 (173.1 to 185.7) | 18918 (18240 to 19571) | 175.8 (169.5 to 182.0) | -2.0% (-3.6 to -0.4) |
| Switzerland | 15449 (14891 to 15997) | 211.8 (204.2 to 218.9) | 19321 (18587 to 20062) | 203.9 (196.9 to 211.5) | -3.7% (-5.6 to -1.7) |
| United Kingdom | 130679 (126486 to 134745) | 218.3 (211.5 to 225.2) | 144237 (139636 to 148769) | 201.6 (195.4 to 207.8) | -7.6% (-8.0 to -7.2) |
| Australasia | 32497 (31226 to 33743) | 157.8 (151.9 to 163.8) | 43553 (41826 to 45339) | 145.4 (139.6 to 151.4) | -7.9% (-9.5 to -6.3) |
| Australia | 26593 (25522 to 27670) | 155.1 (149.1 to 161.1) | 36165 (34649 to 37716) | 142.7 (136.8 to 148.8) | -7.9% (-9.9 to -5.9) |
| New Zealand | 5904 (5695 to 6104) | 171.6 (165.7 to 177.6) | 7387 (7116 to 7647) | 159.9 (154.6 to 165.4) | -6.8% (-8.4 to -5.2) |

Data in parentheses are 95% uncertainty intervals. SDI= Socio-demographic Index.

*Supplementary Table 2*. Prevalence of psoriasis in 1990 and 2019 for both sexes and percentage change of age-standardized rates, by location.

Data in parentheses are 95% uncertainty intervals. SDI= Socio-demographic Index.

| Location | 1990 |  | 2019 |  | Percentage change in age-standardised rates, 1990-2019 |
| --- | --- | --- | --- | --- | --- |
|  | Number | Age-standardized rate  (per 100 000 people) | Number | Age-standardized rate  (per 100 000 people) |  |
| Global | 31585427 (30534514 to 32628378) | 660.2 (637.4 to 681.5) | 40805386 (39421384 to 42076746) | 503.6 (486.9 to 519.2) | -23.7% (-24.0 to -23.5) |
| Low SDI | 1412834 (1363197 to 1462570) | 338.2 (326.6 to 349.3) | 2768895 (2671880 to 2866226) | 300.8 (290.5 to 311.1) | -11.1% (-11.5 to -10.6) |
| Low-middle SDI | 3860144 (3727218 to 3994989) | 409.6 (395.5 to 422.6) | 5936606 (5732917 to 6140587) | 352.1 (340.3 to 363.7) | -14.0% (-14.4 to -13.7) |
| Middle SDI | 6333375 (6115164 to 6558486) | 426.2 (411.0 to 440.2) | 8702298 (8406322 to 8976709) | 338.6 (327.5 to 348.9) | -20.6% (-21.0 to -20.2) |
| High-middle SDI | 8584857 (8275083 to 8879020) | 747.3 (719.8 to 772.5) | 10112276 (9750387 to 10441961) | 589.9 (569.2 to 608.5) | -21.1% (-21.4 to -20.7) |
| High SDI | 11378721 (11002741 to 11726061) | 1255.9 (1214.2 to 1294.7) | 13263867 (12835877 to 13688669) | 1072.7 (1038.7 to 1106.0) | -14.6% (-15.1 to -14.1) |
| Central Sub-Saharan Africa | 223326 (214357 to 232434) | 509.9 (491.1 to 529.4) | 445748 (427754 to 462996) | 412.7 (397.2 to 427.9) | -19.1% (-20.1 to -18.0) |
| Angola | 45459 (43592 to 47366) | 560.0 (538.2 to 581.8) | 98617 (94412 to 102832) | 409.3 (394.0 to 425.8) | -26.9% (-28.3 to -25.4) |
| Central African Republic | 12248 (11755 to 12757) | 556.3 (534.6 to 578.2) | 21137 (20267 to 22051) | 484.1 (465.1 to 503.9) | -13.0% (-14.7 to -11.3) |
| Congo | 8391 (8049 to 8755) | 425.8 (409.5 to 443.3) | 15045 (14427 to 15646) | 322.3 (309.2 to 334.4) | -24.3% (-25.9 to -22.8) |
| Democratic Republic of the Congo | 151930 (145677 to 158313) | 501.2 (481.7 to 520.9) | 302751 (290176 to 315317) | 420.5 (404.4 to 436.4) | -16.1% (-17.6 to -14.4) |
| Equatorial Guinea | 1884 (1809 to 1965) | 557.6 (535.4 to 581.5) | 3464 (3314 to 3622) | 293.9 (282.2 to 305.9) | -47.3% (-48.4 to -46.1) |
| Gabon | 3414 (3280 to 3554) | 409.4 (393.3 to 425.4) | 4735 (4545 to 4918) | 293.7 (282.4 to 305.0) | -28.3% (-29.6 to -26.7) |
| Eastern Sub-Saharan Africa | 269539 (259353 to 280320) | 184.2 (177.6 to 191.0) | 551213 (530511 to 573329) | 166.8 (160.8 to 172.9) | -9.4% (-10.0 to -8.9) |
| Burundi | 7657 (7351 to 7966) | 177.8 (171.3 to 184.3) | 16695 (16066 to 17369) | 178.2 (171.6 to 184.5) | 0.2% (-2.1 to 2.4) |
| Comoros | 639 (615 to 665) | 172.9 (166.5 to 179.3) | 1046 (1007 to 1083) | 158.7 (153.0 to 164.4) | -8.2% (-10.1 to -6.3) |
| Djibouti | 647 (622 to 672) | 173.9 (168.1 to 180.5) | 1667 (1607 to 1731) | 155.3 (150.0 to 160.7) | -10.7% (-12.5 to -9.0) |
| Eritrea | 4500 (4328 to 4679) | 195.7 (188.6 to 202.7) | 9621 (9247 to 9992) | 173.2 (167.0 to 179.2) | -11.5% (-13.3 to -9.5) |
| Ethiopia | 75831 (73181 to 78565) | 192.1 (185.4 to 198.4) | 146872 (141568 to 152534) | 171.5 (165.8 to 177.0) | -10.7% (-11.6 to -9.9) |
| Kenya | 27481 (26504 to 28470) | 156.7 (151.7 to 161.9) | 60934 (58821 to 63192) | 142.9 (138.1 to 147.5) | -8.8% (-9.2 to -8.5) |
| Madagascar | 17537 (16874 to 18222) | 187.1 (180.3 to 193.8) | 38830 (37410 to 40352) | 177.2 (170.9 to 183.5) | -5.3% (-7.3 to -3.4) |
| Malawi | 14367 (13803 to 14905) | 193.5 (186.3 to 200.2) | 26064 (25026 to 27109) | 176.4 (169.7 to 182.7) | -8.9% (-10.7 to -7.0) |
| Mozambique | 22645 (21816 to 23532) | 217.4 (209.4 to 225.6) | 43623 (41974 to 45403) | 192.0 (185.0 to 198.7) | -11.7% (-13.4 to -9.8) |
| Rwanda | 8525 (7996 to 9078) | 153.6 (144.2 to 163.1) | 14860 (13995 to 15826) | 137.4 (129.9 to 145.6) | -10.6% (-13.0 to -8.1) |
| Somalia | 11916 (11446 to 12391) | 216.0 (208.1 to 223.8) | 35007 (33616 to 36450) | 227.6 (219.3 to 236.0) | 5.3% (3.3 to 7.6) |
| South Sudan | 7986 (7666 to 8332) | 173.1 (166.8 to 180.1) | 12311 (11864 to 12785) | 166.5 (160.7 to 172.4) | -3.8% (-5.9 to -1.9) |
| United Republic of Tanzania | 35404 (33086 to 37821) | 176.1 (165.5 to 188.2) | 72052 (67536 to 76936) | 157.8 (148.1 to 168.2) | -10.4% (-12.9 to -7.7) |
| Uganda | 23738 (22830 to 24761) | 182.8 (176.4 to 189.7) | 48929 (47024 to 50862) | 155.7 (150.1 to 161.2) | -14.9% (-16.5 to -13.0) |
| Zambia | 10468 (10077 to 10891) | 173.3 (167.1 to 179.5) | 22262 (21424 to 23150) | 152.7 (147.3 to 157.7) | -11.9% (-13.5 to -10.2) |
| Southern Sub-Saharan Africa | 112241 (108415 to 116162) | 249.1 (240.5 to 257.3) | 168145 (162227 to 174017) | 223.0 (215.2 to 230.2) | -10.5% (-11.0 to -9.8) |
| Botswana | 2748 (2642 to 2858) | 259.1 (249.4 to 268.9) | 4702 (4523 to 4896) | 212.3 (204.7 to 220.4) | -18.1% (-19.7 to -16.3) |
| Lesotho | 4737 (4556 to 4920) | 312.5 (301.0 to 324.3) | 5086 (4891 to 5296) | 263.2 (253.4 to 273.3) | -15.8% (-17.5 to -14.1) |
| Namibia | 2975 (2854 to 3089) | 255.1 (245.2 to 264.3) | 4692 (4510 to 4878) | 217.4 (208.9 to 225.4) | -14.8% (-16.5 to -13.0) |
| South Africa | 78720 (76016 to 81498) | 242.0 (233.9 to 249.9) | 119491 (115279 to 123466) | 216.2 (208.8 to 223.1) | -10.7% (-11.4 to -9.9) |
| Eswatini | 1732 (1664 to 1806) | 277.8 (267.1 to 288.0) | 2354 (2258 to 2450) | 234.4 (225.5 to 243.3) | -15.6% (-17.3 to -13.8) |
| Zimbabwe | 21328 (20506 to 22199) | 262.6 (252.6 to 272.5) | 31820 (30520 to 33180) | 250.1 (240.7 to 259.5) | -4.7% (-6.6 to -2.8) |
| Western Sub-Saharan Africa | 451036 (434831 to 467402) | 292.9 (282.4 to 302.6) | 840876 (810132 to 871926) | 225.2 (217.3 to 232.9) | -23.1% (-23.5 to -22.7) |
| Benin | 9227 (8872 to 9560) | 249.3 (240.5 to 257.3) | 21095 (20304 to 21946) | 210.5 (202.9 to 218.3) | -15.6% (-17.3 to -14.0) |
| Burkina Faso | 20662 (19848 to 21468) | 277.3 (266.2 to 287.2) | 43450 (41744 to 45136) | 241.1 (232.3 to 249.4) | -13.0% (-14.9 to -11.3) |
| Cameroon | 17833 (17179 to 18498) | 216.5 (208.9 to 223.8) | 43551 (41894 to 45218) | 180.3 (173.7 to 186.9) | -16.7% (-18.3 to -15.0) |
| Cabo Verde | 686 (661 to 712) | 238.3 (229.7 to 246.9) | 1021 (982 to 1057) | 186.7 (179.8 to 193.1) | -21.6% (-23.3 to -20.0) |
| Chad | 13563 (13052 to 14081) | 290.6 (279.6 to 300.5) | 30717 (29507 to 31976) | 250.6 (241.3 to 259.5) | -13.8% (-15.6 to -12.0) |
| Cote d'Ivoire | 22056 (21232 to 22925) | 232.5 (224.4 to 240.4) | 43144 (41561 to 44866) | 197.1 (190.1 to 204.0) | -15.2% (-17.0 to -13.5) |
| Gambia | 1969 (1894 to 2047) | 258.1 (249.0 to 267.3) | 3911 (3763 to 4055) | 211.3 (204.0 to 218.2) | -18.1% (-19.8 to -16.6) |
| Ghana | 33425 (31545 to 35459) | 278.2 (263.0 to 294.5) | 63901 (60284 to 67619) | 230.4 (217.1 to 243.3) | -17.2% (-19.6 to -15.0) |
| Guinea | 13088 (12596 to 13587) | 261.8 (251.9 to 271.3) | 22811 (21970 to 23679) | 225.1 (217.1 to 233.6) | -14.0% (-15.8 to -12.3) |
| Guinea-Bissau | 2066 (1988 to 2143) | 262.9 (253.1 to 272.0) | 3437 (3305 to 3570) | 221.1 (212.8 to 228.9) | -15.9% (-17.5 to -14.2) |
| Liberia | 3858 (3720 to 4005) | 237.9 (229.4 to 246.1) | 8448 (8130 to 8793) | 206.6 (199.1 to 213.9) | -13.2% (-15.0 to -11.4) |
| Mali | 15907 (14987 to 16938) | 230.9 (217.6 to 244.4) | 32172 (30136 to 34268) | 188.4 (177.5 to 200.7) | -18.4% (-20.6 to -16.0) |
| Mauritania | 3996 (3845 to 4149) | 240.7 (231.6 to 249.6) | 6865 (6595 to 7134) | 200.5 (193.0 to 207.8) | -16.7% (-18.4 to -14.9) |
| Niger | 18256 (17544 to 18999) | 302.4 (291.7 to 313.3) | 47696 (45816 to 49648) | 277.2 (267.3 to 286.6) | -8.3% (-10.2 to -6.5) |
| Nigeria | 245222 (236924 to 253676) | 332.2 (321.0 to 342.8) | 412338 (398054 to 426906) | 233.7 (225.8 to 241.2) | -29.6% (-30.1 to -29.2) |
| Sao Tome and Principe | 207 (200 to 215) | 212.1 (204.5 to 219.8) | 314 (303 to 326) | 172.3 (166.1 to 178.6) | -18.7% (-20.3 to -17.1) |
| Senegal | 15004 (14447 to 15621) | 252.5 (243.6 to 261.3) | 27632 (26615 to 28664) | 216.0 (208.5 to 223.8) | -14.4% (-16.2 to -12.7) |
| Sierra Leone | 7509 (7218 to 7785) | 250.4 (241.3 to 258.8) | 14917 (14309 to 15510) | 216.7 (208.6 to 224.6) | -13.4% (-15.1 to -11.6) |
| Togo | 6485 (6241 to 6757) | 231.7 (223.5 to 240.0) | 13443 (12957 to 13958) | 198.3 (191.4 to 205.6) | -14.4% (-16.1 to -12.7) |
| Andean Latin America | 275169 (264742 to 285945) | 851.5 (819.5 to 883.6) | 444522 (427695 to 461805) | 712.9 (685.9 to 739.6) | -16.3% (-17.3 to -15.2) |
| Bolivia (Plurinational State of) | 53520 (51382 to 55765) | 1015.5 (976.6 to 1056.9) | 93668 (89881 to 97528) | 840.2 (805.7 to 873.5) | -17.3% (-18.9 to -15.7) |
| Ecuador | 66163 (63454 to 68751) | 777.3 (747.3 to 805.8) | 110320 (105995 to 114553) | 642.4 (617.2 to 666.7) | -17.4% (-18.9 to -15.7) |
| Peru | 155486 (149120 to 161635) | 839.3 (806.3 to 872.1) | 240535 (230423 to 250080) | 707.2 (678.3 to 734.2) | -15.7% (-17.3 to -14.2) |
| Tropical Latin America | 1098728 (1061487 to 1136829) | 808.9 (781.5 to 835.6) | 1830253 (1765446 to 1893664) | 767.2 (741.1 to 792.9) | -5.2% (-5.7 to -4.6) |
| Brazil | 1070145 (1033787 to 1107136) | 807.9 (780.5 to 834.5) | 1775848 (1713032 to 1836953) | 765.9 (739.7 to 791.4) | -5.2% (-5.8 to -4.7) |
| Paraguay | 28583 (27440 to 29705) | 848.8 (813.8 to 880.9) | 54404 (52185 to 56716) | 812.8 (780.0 to 846.6) | -4.2% (-6.0 to -2.4) |
| Central Latin America | 211206 (203949 to 218501) | 150.6 (145.7 to 155.6) | 334104 (322899 to 345848) | 132.0 (127.7 to 136.5) | -12.3% (-12.8 to -11.8) |
| Colombia | 39218 (37713 to 40620) | 137.1 (132.0 to 141.9) | 59687 (57551 to 62070) | 119.3 (115.1 to 123.9) | -13.0% (-14.6 to -11.4) |
| Costa Rica | 3581 (3435 to 3720) | 134.6 (129.5 to 139.6) | 5901 (5672 to 6132) | 118.2 (113.6 to 122.6) | -12.2% (-13.6 to -10.6) |
| El Salvador | 7148 (6888 to 7411) | 159.7 (153.9 to 165.5) | 8325 (8019 to 8648) | 135.2 (130.2 to 140.3) | -15.3% (-16.8 to -13.8) |
| Guatemala | 11500 (11071 to 11956) | 178.7 (172.5 to 185.1) | 23372 (22554 to 24275) | 145.5 (140.3 to 150.8) | -18.5% (-20.2 to -16.8) |
| Honduras | 6547 (6282 to 6811) | 174.8 (168.6 to 181.4) | 13368 (12879 to 13891) | 150.6 (145.2 to 156.4) | -13.9% (-15.6 to -12.2) |
| Mexico | 113131 (109250 to 117069) | 155.5 (150.5 to 160.6) | 173194 (167265 to 179098) | 136.2 (131.7 to 140.7) | -12.4% (-12.9 to -12.0) |
| Nicaragua | 5138 (4942 to 5347) | 168.4 (162.2 to 174.6) | 8752 (8428 to 9089) | 142.4 (137.0 to 147.8) | -15.4% (-17.0 to -13.7) |
| Panama | 2808 (2699 to 2925) | 131.2 (126.3 to 136.3) | 4879 (4694 to 5065) | 116.5 (112.0 to 120.8) | -11.3% (-13.0 to -9.5) |
| Venezuela (Bolivarian Republic of) | 22135 (21288 to 23061) | 136.1 (131.0 to 141.2) | 36627 (35310 to 38073) | 124.7 (120.2 to 129.6) | -8.4% (-10.0 to -6.5) |
| Southern Latin America | 499330 (480104 to 518209) | 1032.1 (992.4 to 1071.2) | 650187 (624126 to 674258) | 898.7 (863.3 to 933.3) | -12.9% (-14.1 to -11.7) |
| Argentina | 336654 (323442 to 350364) | 1031.6 (991.3 to 1073.6) | 436613 (419337 to 453631) | 909.5 (873.4 to 945.8) | -11.8% (-13.4 to -10.2) |
| Chile | 129240 (124069 to 134166) | 1039.6 (999.6 to 1077.9) | 178844 (171541 to 185813) | 874.0 (839.4 to 907.8) | -15.9% (-17.5 to -14.3) |
| Uruguay | 33416 (32113 to 34706) | 1009.4 (970.5 to 1049.1) | 34698 (33292 to 35945) | 894.7 (858.3 to 928.2) | -11.4% (-13.0 to -9.5) |
| Caribbean | 145256 (140066 to 150288) | 446.4 (430.8 to 461.6) | 202492 (195171 to 209683) | 413.1 (398.4 to 427.7) | -7.5% (-8.3 to -6.6) |
| Antigua and Barbuda | 223 (214 to 231) | 390.8 (376.0 to 405.0) | 343 (328 to 356) | 349.7 (336.0 to 362.3) | -10.5% (-12.3 to -8.7) |
| Bahamas | 874 (841 to 910) | 373.8 (359.9 to 387.9) | 1413 (1357 to 1464) | 346.9 (334.4 to 359.6) | -7.2% (-9.0 to -5.3) |
| Barbados | 930 (897 to 964) | 362.1 (348.8 to 374.8) | 1192 (1148 to 1236) | 338.3 (325.7 to 350.3) | -6.6% (-8.5 to -4.7) |
| Belize | 747 (718 to 775) | 497.3 (478.1 to 515.1) | 1590 (1529 to 1651) | 411.4 (396.3 to 426.2) | -17.3% (-19.0 to -15.6) |
| Bermuda | 254 (244 to 264) | 400.0 (385.3 to 414.6) | 287 (276 to 298) | 358.6 (345.4 to 372.4) | -10.4% (-12.3 to -8.5) |
| Cuba | 45454 (43756 to 47138) | 418.0 (402.1 to 433.6) | 53258 (51082 to 55351) | 392.9 (377.0 to 407.4) | -6.0% (-7.8 to -4.1) |
| Dominica | 279 (269 to 289) | 403.6 (388.7 to 418.0) | 265 (255 to 275) | 352.3 (339.2 to 364.5) | -12.7% (-14.5 to -10.9) |
| Dominican Republic | 29668 (28480 to 30857) | 488.4 (469.8 to 506.3) | 44463 (42759 to 46147) | 417.4 (401.6 to 432.8) | -14.5% (-16.2 to -12.9) |
| Grenada | 340 (328 to 353) | 446.1 (429.2 to 462.8) | 409 (394 to 425) | 369.6 (356.4 to 383.4) | -17.2% (-18.7 to -15.5) |
| Guyana | 2737 (2629 to 2839) | 416.9 (401.0 to 432.2) | 2790 (2679 to 2895) | 367.1 (352.4 to 380.3) | -11.9% (-13.8 to -10.1) |
| Haiti | 29642 (28493 to 30748) | 563.3 (541.8 to 584.2) | 55799 (53465 to 57965) | 504.3 (484.9 to 523.1) | -10.5% (-12.2 to -8.6) |
| Jamaica | 9132 (8799 to 9487) | 432.2 (416.0 to 448.9) | 11021 (10591 to 11439) | 375.6 (361.4 to 389.7) | -13.1% (-14.9 to -11.4) |
| Puerto Rico | 13212 (12721 to 13682) | 364.0 (350.5 to 377.3) | 13739 (13209 to 14294) | 324.4 (312.8 to 336.4) | -10.9% (-12.6 to -9.1) |
| Saint Kitts and Nevis | 152 (146 to 158) | 398.0 (383.3 to 412.6) | 229 (220 to 238) | 346.0 (333.3 to 358.1) | -13.1% (-14.9 to -11.2) |
| Saint Lucia | 509 (490 to 529) | 429.3 (413.1 to 444.7) | 724 (696 to 751) | 370.9 (356.4 to 384.2) | -13.6% (-15.3 to -11.9) |
| Saint Vincent and the Grenadines | 426 (410 to 442) | 446.2 (429.0 to 463.0) | 474 (455 to 493) | 385.5 (370.7 to 400.1) | -13.6% (-15.3 to -11.8) |
| Suriname | 1399 (1347 to 1453) | 399.0 (383.8 to 414.0) | 2140 (2057 to 2219) | 355.3 (342.2 to 367.9) | -10.9% (-12.7 to -9.1) |
| Trinidad and Tobago | 4053 (3903 to 4212) | 366.2 (352.4 to 379.3) | 5100 (4908 to 5296) | 325.0 (312.9 to 336.5) | -11.2% (-13.0 to -9.2) |
| United States Virgin Islands | 387 (372 to 401) | 370.8 (357.1 to 384.3) | 393 (378 to 408) | 317.9 (306.3 to 329.2) | -14.3% (-16.1 to -12.5) |
| Central Europe | 722895 (696262 to 746321) | 538.3 (518.3 to 555.7) | 624819 (606034 to 641226) | 440.8 (428.2 to 452.1) | -18.1% (-19.0 to -17.2) |
| Albania | 17262 (16583 to 17903) | 585.4 (563.1 to 606.1) | 14473 (13929 to 14992) | 447.7 (431.4 to 463.5) | -23.5% (-25.1 to -21.8) |
| Bosnia and Herzegovina | 28350 (27219 to 29366) | 600.3 (577.2 to 620.8) | 18316 (17578 to 18986) | 438.8 (421.8 to 454.3) | -26.9% (-28.4 to -25.4) |
| Bulgaria | 53888 (51837 to 55805) | 529.7 (510.4 to 547.3) | 36772 (35328 to 38115) | 415.8 (400.2 to 430.0) | -21.5% (-23.1 to -19.9) |
| Croatia | 28662 (27497 to 29697) | 511.1 (491.1 to 529.3) | 21910 (21088 to 22727) | 409.3 (394.3 to 423.7) | -19.9% (-21.4 to -18.3) |
| Czechia | 60295 (58031 to 62417) | 521.9 (502.3 to 539.8) | 53744 (51592 to 55697) | 406.6 (391.4 to 420.5) | -22.1% (-23.6 to -20.4) |
| Hungary | 63507 (61050 to 65558) | 531.3 (511.6 to 548.7) | 50493 (48496 to 52342) | 415.1 (399.5 to 429.2) | -21.9% (-23.5 to -20.2) |
| North Macedonia | 11041 (10620 to 11427) | 536.2 (515.9 to 554.7) | 11002 (10580 to 11384) | 421.5 (405.8 to 436.1) | -21.4% (-23.0 to -19.8) |
| Montenegro | 3173 (3057 to 3286) | 490.9 (472.9 to 507.7) | 2994 (2876 to 3101) | 402.4 (386.7 to 416.0) | -18.0% (-19.7 to -16.4) |
| Poland | 229064 (219825 to 237321) | 562.5 (540.7 to 582.5) | 239140 (234147 to 243637) | 503.2 (493.3 to 512.5) | -10.6% (-12.6 to -8.3) |
| Romania | 129849 (124860 to 134196) | 507.3 (488.3 to 524.4) | 93518 (89990 to 96898) | 391.5 (377.3 to 405.1) | -22.8% (-24.4 to -21.2) |
| Serbia | 56883 (54697 to 59012) | 539.7 (519.0 to 558.7) | 44926 (43178 to 46564) | 419.1 (403.4 to 433.1) | -22.3% (-24.0 to -20.6) |
| Slovakia | 30182 (29059 to 31207) | 540.5 (520.3 to 559.2) | 27342 (26275 to 28302) | 409.0 (394.5 to 423.3) | -24.3% (-25.9 to -22.8) |
| Slovenia | 10737 (10333 to 11113) | 490.3 (472.4 to 507.3) | 10187 (9763 to 10570) | 391.5 (377.1 to 405.1) | -20.2% (-21.8 to -18.4) |
| Eastern Europe | 1213698 (1169429 to 1257821) | 486.8 (469.1 to 503.5) | 1072655 (1036778 to 1105301) | 423.7 (410.5 to 436.8) | -13.0% (-13.7 to -12.2) |
| Belarus | 64208 (61747 to 66495) | 559.0 (537.5 to 579.0) | 50818 (48786 to 52696) | 439.4 (423.4 to 454.8) | -21.4% (-23.0 to -19.7) |
| Estonia | 9220 (8861 to 9544) | 528.9 (509.0 to 547.3) | 6653 (6404 to 6889) | 416.4 (401.1 to 430.0) | -21.3% (-22.9 to -19.5) |
| Latvia | 15480 (14893 to 16031) | 518.7 (499.5 to 536.7) | 9839 (9454 to 10208) | 415.7 (399.9 to 430.2) | -19.9% (-21.5 to -18.2) |
| Lithuania | 20700 (19886 to 21453) | 517.1 (497.1 to 535.8) | 13971 (13441 to 14485) | 403.3 (388.7 to 417.0) | -22.0% (-23.6 to -20.4) |
| Republic of Moldova | 24491 (23580 to 25420) | 537.8 (518.0 to 557.9) | 19901 (19116 to 20631) | 443.6 (427.1 to 460.3) | -17.5% (-19.2 to -15.7) |
| Russian Federation | 779825 (750010 to 808710) | 472.7 (454.8 to 489.6) | 737320 (713319 to 759173) | 420.4 (407.6 to 432.7) | -11.1% (-11.9 to -10.2) |
| Ukraine | 299774 (288598 to 310700) | 502.7 (484.2 to 521.3) | 234154 (225386 to 242681) | 431.3 (416.3 to 446.9) | -14.2% (-15.9 to -12.5) |
| North Africa and Middle East | 1587747 (1531963 to 1645830) | 566.5 (545.7 to 586.0) | 2426320 (2342733 to 2509856) | 414.3 (400.3 to 427.8) | -26.9% (-27.5 to -26.2) |
| Afghanistan | 66198 (63700 to 68702) | 716.9 (688.5 to 743.9) | 164532 (158229 to 171117) | 580.0 (559.3 to 601.7) | -19.1% (-20.6 to -17.5) |
| Algeria | 102650 (98636 to 106681) | 503.4 (483.4 to 521.9) | 161545 (155214 to 167945) | 387.8 (373.3 to 402.0) | -23.0% (-24.7 to -21.4) |
| Bahrain | 1921 (1841 to 2006) | 427.3 (411.7 to 443.4) | 5734 (5479 to 5992) | 344.5 (331.8 to 357.1) | -19.4% (-21.1 to -17.6) |
| Egypt | 128397 (123537 to 133646) | 271.8 (261.6 to 281.8) | 195678 (188818 to 203095) | 212.8 (205.5 to 220.5) | -21.7% (-23.4 to -20.0) |
| Iran (Islamic Republic of) | 251002 (242400 to 259481) | 539.8 (521.4 to 557.2) | 342121 (329864 to 353823) | 387.8 (374.5 to 400.3) | -28.1% (-28.6 to -27.6) |
| Iraq | 85726 (82467 to 89057) | 636.2 (610.5 to 660.1) | 172419 (165642 to 179123) | 460.8 (443.5 to 478.0) | -27.6% (-29.1 to -26.2) |
| Jordan | 13562 (13049 to 14102) | 469.6 (452.3 to 486.8) | 38626 (37226 to 40110) | 362.4 (349.2 to 375.2) | -22.8% (-24.5 to -21.1) |
| Kuwait | 5959 (5713 to 6215) | 380.5 (366.2 to 394.7) | 14901 (14267 to 15532) | 312.4 (300.5 to 323.7) | -17.9% (-19.6 to -16.1) |
| Lebanon | 14559 (13977 to 15091) | 503.2 (482.6 to 521.3) | 19691 (18928 to 20428) | 372.0 (357.4 to 385.7) | -26.1% (-27.5 to -24.5) |
| Libya | 15592 (15006 to 16185) | 468.8 (450.5 to 485.8) | 24353 (23400 to 25268) | 347.4 (334.5 to 359.5) | -25.9% (-27.4 to -24.2) |
| Morocco | 125039 (119954 to 129926) | 587.0 (564.4 to 609.0) | 165802 (159408 to 171800) | 453.8 (436.9 to 469.7) | -22.7% (-24.2 to -21.1) |
| Palestine | 9253 (8886 to 9604) | 605.7 (582.7 to 628.0) | 17506 (16828 to 18175) | 418.0 (402.4 to 432.8) | -31.0% (-32.3 to -29.5) |
| Oman | 7698 (7390 to 8016) | 493.5 (474.6 to 511.5) | 14214 (13582 to 14826) | 315.2 (303.6 to 326.3) | -36.1% (-37.6 to -34.7) |
| Qatar | 1677 (1607 to 1750) | 406.8 (391.8 to 421.6) | 9310 (8909 to 9749) | 304.8 (293.2 to 315.3) | -25.1% (-26.8 to -23.3) |
| Saudi Arabia | 69025 (66128 to 71777) | 539.3 (517.5 to 559.4) | 118143 (113105 to 122798) | 318.8 (306.8 to 329.3) | -40.9% (-42.3 to -39.4) |
| Sudan | 91509 (87967 to 94909) | 583.5 (560.1 to 606.1) | 139454 (134285 to 144946) | 413.8 (398.8 to 429.4) | -29.1% (-30.6 to -27.6) |
| Syrian Arab Republic | 54652 (52530 to 56839) | 561.3 (539.2 to 582.0) | 60768 (58411 to 62946) | 421.7 (405.7 to 436.7) | -24.9% (-26.3 to -23.3) |
| Tunisia | 37648 (36230 to 39170) | 518.5 (498.2 to 538.1) | 49550 (47665 to 51337) | 394.5 (379.5 to 408.5) | -23.9% (-25.4 to -22.3) |
| Turkey | 436410 (419077 to 454487) | 841.1 (806.6 to 873.8) | 561800 (539319 to 582297) | 628.3 (603.8 to 650.0) | -25.3% (-26.9 to -23.6) |
| United Arab Emirates | 6520 (6224 to 6820) | 390.6 (375.8 to 404.3) | 31374 (29786 to 32925) | 288.5 (277.8 to 298.1) | -26.1% (-27.8 to -24.4) |
| Yemen | 61682 (59225 to 64004) | 624.8 (600.0 to 647.8) | 116335 (111877 to 120793) | 457.6 (440.5 to 474.3) | -26.8% (-28.3 to -25.3) |
| Central Asia | 346729 (333733 to 359973) | 567.8 (546.1 to 588.6) | 420455 (404188 to 436205) | 454.9 (437.6 to 471.2) | -19.9% (-20.6 to -19.2) |
| Armenia | 18196 (17453 to 18888) | 555.7 (533.8 to 576.5) | 14642 (14065 to 15207) | 423.4 (407.5 to 439.2) | -23.8% (-25.4 to -22.2) |
| Azerbaijan | 34845 (33466 to 36216) | 525.1 (505.1 to 545.4) | 47567 (45550 to 49507) | 430.3 (413.1 to 446.5) | -18.1% (-19.7 to -16.3) |
| Georgia | 29258 (28112 to 30390) | 497.9 (479.0 to 516.1) | 18391 (17638 to 19162) | 429.5 (411.9 to 447.0) | -13.7% (-15.5 to -11.8) |
| Kazakhstan | 85920 (82400 to 89412) | 557.0 (534.1 to 579.4) | 83045 (79678 to 86467) | 440.7 (423.7 to 458.2) | -20.9% (-22.5 to -19.2) |
| Kyrgyzstan | 22403 (21530 to 23296) | 580.0 (556.8 to 603.5) | 29965 (28745 to 31201) | 490.9 (471.3 to 510.2) | -15.4% (-17.1 to -13.5) |
| Mongolia | 10711 (10263 to 11150) | 627.2 (601.8 to 651.2) | 15772 (15158 to 16372) | 482.9 (465.0 to 499.8) | -23.0% (-24.6 to -21.5) |
| Tajikistan | 25843 (24750 to 26862) | 613.7 (588.5 to 637.5) | 42469 (40706 to 44245) | 504.5 (484.7 to 523.9) | -17.8% (-19.4 to -16.1) |
| Turkmenistan | 16621 (15969 to 17279) | 553.0 (532.1 to 573.2) | 20587 (19803 to 21409) | 416.9 (401.3 to 432.6) | -24.6% (-26.2 to -23.0) |
| Uzbekistan | 102931 (98521 to 106958) | 607.8 (582.4 to 631.1) | 148018 (141953 to 153598) | 463.3 (445.1 to 479.7) | -23.8% (-25.3 to -22.1) |
| South Asia | 3311089 (3196934 to 3427452) | 352.6 (340.5 to 363.8) | 5873201 (5668229 to 6075556) | 334.5 (322.9 to 345.6) | -5.1% (-5.6 to -4.7) |
| Bangladesh | 293760 (282673 to 305375) | 336.4 (324.0 to 348.3) | 435327 (418741 to 451924) | 277.6 (267.1 to 287.4) | -17.5% (-19.1 to -15.8) |
| Bhutan | 1777 (1709 to 1845) | 357.9 (344.5 to 369.7) | 2206 (2119 to 2294) | 298.3 (286.8 to 309.7) | -16.6% (-18.4 to -15.0) |
| India | 2642355 (2551537 to 2734222) | 355.1 (342.9 to 366.5) | 4798113 (4629240 to 4964355) | 348.3 (336.0 to 360.0) | -1.9% (-2.4 to -1.5) |
| Nepal | 51456 (49467 to 53296) | 320.1 (308.7 to 331.1) | 67611 (65130 to 69934) | 234.4 (226.1 to 242.0) | -26.8% (-28.4 to -25.2) |
| Pakistan | 321740 (310707 to 332941) | 353.0 (340.8 to 364.5) | 569944 (550288 to 590159) | 296.3 (285.7 to 306.0) | -16.1% (-17.1 to -15.0) |
| Southeast Asia | 585643 (565704 to 606242) | 150.2 (145.2 to 155.3) | 887599 (856929 to 918713) | 128.8 (124.5 to 133.2) | -14.3% (-14.8 to -13.7) |
| Cambodia | 16383 (15778 to 17043) | 211.2 (203.2 to 218.7) | 27044 (26025 to 28074) | 175.4 (169.0 to 181.6) | -17.0% (-18.7 to -15.2) |
| Indonesia | 137144 (132204 to 142147) | 88.6 (85.6 to 91.6) | 210973 (203268 to 218987) | 79.8 (76.9 to 82.7) | -9.9% (-10.5 to -9.3) |
| Lao People's Democratic Republic | 7098 (6830 to 7369) | 219.2 (211.2 to 227.2) | 11596 (11159 to 12054) | 180.8 (174.3 to 187.3) | -17.5% (-19.2 to -15.8) |
| Malaysia | 21372 (20551 to 22273) | 144.9 (139.5 to 150.3) | 37967 (36450 to 39536) | 121.0 (116.4 to 125.9) | -16.5% (-18.1 to -14.9) |
| Maldives | 302 (291 to 313) | 184.7 (178.1 to 190.8) | 701 (674 to 730) | 143.7 (138.8 to 148.8) | -22.2% (-23.9 to -20.4) |
| Mauritius | 1677 (1617 to 1742) | 166.4 (160.5 to 172.4) | 2149 (2064 to 2234) | 142.5 (137.2 to 148.1) | -14.4% (-16.2 to -12.6) |
| Myanmar | 73120 (70574 to 75992) | 214.6 (207.1 to 222.4) | 94290 (90857 to 97724) | 174.5 (168.5 to 180.5) | -18.7% (-20.3 to -17.0) |
| Philippines | 86656 (83753 to 89614) | 171.3 (165.7 to 176.6) | 161685 (156141 to 167074) | 157.0 (151.8 to 162.1) | -8.3% (-8.7 to -7.9) |
| Sri Lanka | 49385 (47151 to 51672) | 322.3 (307.6 to 337.4) | 60874 (58019 to 63691) | 254.4 (242.7 to 266.1) | -21.1% (-22.8 to -19.3) |
| Seychelles | 95 (92 to 99) | 144.7 (139.5 to 150.3) | 143 (138 to 149) | 127.6 (123.0 to 132.4) | -11.8% (-13.5 to -10.0) |
| Thailand | 83971 (80870 to 87334) | 162.4 (156.4 to 168.4) | 119871 (115194 to 124693) | 140.1 (135.0 to 145.2) | -13.7% (-15.3 to -12.1) |
| Timor-Leste | 1213 (1168 to 1259) | 205.3 (198.0 to 212.5) | 1852 (1785 to 1921) | 165.6 (159.6 to 171.5) | -19.3% (-21.0 to -17.5) |
| Viet Nam | 106450 (102413 to 110531) | 190.1 (183.1 to 197.1) | 157289 (151281 to 163445) | 153.6 (148.1 to 159.2) | -19.2% (-20.9 to -17.3) |
| East Asia | 6579854 (6339592 to 6812721) | 579.5 (558.1 to 599.0) | 7948327 (7669027 to 8215035) | 436.6 (422.0 to 450.1) | -24.7% (-25.1 to -24.2) |
| China | 6335475 (6103054 to 6560476) | 577.9 (556.6 to 597.3) | 7653268 (7385811 to 7908309) | 434.8 (420.3 to 448.3) | -24.8% (-25.2 to -24.3) |
| Democratic People's Republic of Korea | 140626 (134811 to 145984) | 715.3 (686.5 to 741.8) | 175948 (168804 to 182841) | 581.9 (558.8 to 603.7) | -18.7% (-20.2 to -17.1) |
| Taiwan (Province of China) | 103753 (99719 to 107798) | 530.4 (509.4 to 549.5) | 119111 (114398 to 123485) | 390.3 (376.1 to 403.6) | -26.4% (-27.9 to -24.9) |
| Oceania | 15493 (14933 to 16075) | 306.0 (295.1 to 317.2) | 31161 (29970 to 32394) | 278.0 (267.9 to 288.3) | -9.2% (-10.5 to -7.8) |
| American Samoa | 92 (89 to 96) | 239.1 (230.2 to 247.8) | 114 (110 to 118) | 211.0 (203.5 to 218.7) | -11.8% (-13.6 to -10.0) |
| Federated States of Micronesia | 211 (203 to 219) | 271.1 (260.7 to 281.2) | 222 (212 to 230) | 234.3 (225.2 to 242.6) | -13.6% (-15.5 to -11.6) |
| Cook Islands | 39 (38 to 41) | 237.9 (229.1 to 246.8) | 42 (40 to 43) | 204.7 (197.4 to 211.9) | -14.0% (-15.7 to -12.0) |
| Nauru | 18 (17 to 19) | 232.3 (223.7 to 240.9) | 18 (17 to 18) | 206.3 (198.3 to 213.9) | -11.2% (-13.0 to -9.3) |
| Niue | 5 (5 to 6) | 251.6 (241.9 to 260.8) | 4 (4 to 4) | 215.2 (207.2 to 223.0) | -14.4% (-16.2 to -12.6) |
| Palau | 30 (29 to 32) | 221.7 (213.5 to 230.1) | 42 (40 to 44) | 196.3 (188.8 to 203.6) | -11.5% (-13.4 to -9.7) |
| Fiji | 1676 (1613 to 1742) | 268.5 (258.6 to 278.4) | 2067 (1991 to 2142) | 231.4 (223.3 to 239.9) | -13.8% (-15.6 to -12.1) |
| Guam | 258 (248 to 268) | 213.4 (205.6 to 221.2) | 336 (323 to 349) | 187.7 (180.7 to 194.9) | -12.0% (-13.9 to -10.3) |
| Kiribati | 159 (153 to 165) | 268.9 (259.3 to 278.7) | 264 (254 to 275) | 257.6 (247.7 to 267.5) | -4.2% (-6.1 to -2.2) |
| Marshall Islands | 94 (90 to 98) | 293.3 (282.2 to 304.1) | 126 (121 to 131) | 245.7 (236.4 to 255.0) | -16.2% (-17.9 to -14.4) |
| Northern Mariana Islands | 87 (83 to 90) | 217.9 (209.9 to 226.0) | 104 (100 to 109) | 204.5 (196.8 to 212.3) | -6.1% (-8.2 to -4.2) |
| Papua New Guinea | 10206 (9818 to 10618) | 322.2 (310.1 to 334.5) | 23409 (22496 to 24367) | 289.5 (278.7 to 300.4) | -10.2% (-12.0 to -8.4) |
| Samoa | 337 (325 to 350) | 260.2 (250.7 to 269.2) | 469 (451 to 486) | 249.7 (240.2 to 258.4) | -4.0% (-6.0 to -2.1) |
| Solomon Islands | 818 (787 to 850) | 332.8 (320.1 to 344.7) | 1529 (1470 to 1590) | 289.6 (278.6 to 300.4) | -13.0% (-14.7 to -11.2) |
| Tokelau | 4 (4 to 4) | 260.4 (250.5 to 270.5) | 3 (3 to 3) | 216.8 (209.0 to 225.0) | -16.7% (-18.5 to -14.9) |
| Tonga | 218 (210 to 226) | 280.1 (269.6 to 290.3) | 226 (217 to 234) | 245.4 (235.6 to 254.4) | -12.4% (-14.2 to -10.6) |
| Tuvalu | 22 (22 to 23) | 269.9 (259.7 to 279.2) | 26 (25 to 27) | 229.5 (220.7 to 237.9) | -15.0% (-16.8 to -13.2) |
| Vanuatu | 360 (346 to 374) | 317.5 (305.2 to 329.0) | 691 (663 to 717) | 279.1 (268.2 to 289.8) | -12.1% (-13.8 to -10.2) |
| High-income Asia Pacific | 519958 (502481 to 536524) | 274.1 (265.1 to 282.9) | 613404 (591525 to 633631) | 262.2 (253.4 to 270.8) | -4.3% (-5.0 to -3.6) |
| Brunei Darussalam | 531 (511 to 552) | 238.7 (230.6 to 247.6) | 993 (956 to 1036) | 218.7 (210.9 to 227.3) | -8.4% (-10.2 to -6.5) |
| Japan | 383428 (370252 to 395211) | 267.6 (258.9 to 276.0) | 427245 (412685 to 441402) | 266.4 (257.8 to 275.0) | -0.4% (-0.8 to 0.0) |
| Singapore | 7045 (6775 to 7340) | 227.8 (219.9 to 236.3) | 13991 (13443 to 14532) | 205.4 (198.0 to 213.0) | -9.8% (-11.5 to -7.9) |
| Republic of Korea | 128955 (124007 to 133567) | 298.6 (287.9 to 308.4) | 171175 (164493 to 177834) | 259.4 (249.9 to 269.1) | -13.1% (-15.1 to -11.1) |
| High-income North America | 3852420 (3731799 to 3968382) | 1271.7 (1233.3 to 1310.9) | 4693639 (4553019 to 4837451) | 1081.6 (1048.9 to 1115.4) | -14.9% (-15.8 to -14.0) |
| Canada | 343994 (332131 to 355625) | 1161.8 (1122.5 to 1200.4) | 460851 (445254 to 477734) | 1028.5 (996.5 to 1063.8) | -11.5% (-13.1 to -9.6) |
| Greenland | 775 (748 to 803) | 1464.4 (1415.3 to 1510.8) | 808 (781 to 836) | 1276.6 (1235.0 to 1317.1) | -12.8% (-14.5 to -11.1) |
| United States of America | 3507563 (3399207 to 3612354) | 1283.6 (1244.6 to 1323.5) | 4231906 (4106377 to 4360602) | 1087.6 (1055.8 to 1120.3) | -15.3% (-16.2 to -14.3) |
| Western Europe | 9193334 (8851492 to 9512106) | 2080.1 (2005.3 to 2151.1) | 10236919 (9862029 to 10589120) | 1884.1 (1817.4 to 1948.3) | -9.4% (-10.0 to -8.9) |
| Andorra | 1171 (1126 to 1211) | 1952.7 (1881.0 to 2015.6) | 1935 (1860 to 2007) | 1817.1 (1754.0 to 1883.8) | -6.9% (-8.7 to -5.0) |
| Austria | 196998 (189947 to 203977) | 2197.3 (2118.7 to 2278.0) | 220404 (212415 to 228066) | 1972.3 (1900.3 to 2041.2) | -10.2% (-11.9 to -8.4) |
| Belgium | 262949 (253376 to 272337) | 2265.0 (2183.6 to 2345.5) | 281838 (271481 to 291821) | 2013.9 (1939.8 to 2081.3) | -11.1% (-12.8 to -9.3) |
| Cyprus | 17732 (17068 to 18388) | 2191.0 (2110.1 to 2270.5) | 28385 (27275 to 29449) | 1809.6 (1741.3 to 1874.7) | -17.4% (-19.0 to -15.8) |
| Denmark | 132436 (127897 to 137097) | 2210.0 (2131.7 to 2291.4) | 142787 (137755 to 147946) | 2002.7 (1933.3 to 2076.6) | -9.4% (-11.0 to -7.7) |
| Finland | 142559 (137299 to 147583) | 2470.4 (2380.3 to 2557.4) | 154102 (148100 to 159636) | 2216.8 (2133.7 to 2299.1) | -10.3% (-11.8 to -8.5) |
| France | 1821541 (1743204 to 1893730) | 2813.0 (2691.2 to 2927.6) | 2019988 (1934585 to 2103793) | 2503.8 (2395.4 to 2608.6) | -11.0% (-12.9 to -9.2) |
| Germany | 1520302 (1464710 to 1576473) | 1603.2 (1547.9 to 1662.2) | 1622278 (1559949 to 1681992) | 1498.0 (1442.3 to 1553.3) | -6.6% (-8.3 to -4.7) |
| Greece | 267614 (257847 to 276667) | 2232.0 (2151.1 to 2309.7) | 259516 (248883 to 268220) | 1964.3 (1891.2 to 2032.4) | -12.0% (-13.7 to -10.2) |
| Iceland | 6445 (6206 to 6670) | 2463.8 (2371.2 to 2548.6) | 8721 (8401 to 9019) | 2175.6 (2094.8 to 2251.1) | -11.7% (-13.4 to -10.1) |
| Ireland | 86410 (83380 to 89419) | 2349.1 (2266.4 to 2433.2) | 114579 (110402 to 118641) | 2013.1 (1940.0 to 2084.2) | -14.3% (-15.9 to -12.7) |
| Israel | 97301 (93844 to 100589) | 2033.7 (1961.1 to 2102.8) | 175987 (169633 to 182182) | 1830.8 (1762.7 to 1896.5) | -10.0% (-11.7 to -8.2) |
| Italy | 1368628 (1322165 to 1413536) | 2033.2 (1965.0 to 2097.3) | 1491840 (1440844 to 1544404) | 1891.2 (1826.2 to 1954.0) | -7.0% (-7.6 to -6.4) |
| Luxembourg | 9204 (8858 to 9535) | 2073.8 (1997.2 to 2147.1) | 14334 (13809 to 14855) | 1914.9 (1846.0 to 1982.6) | -7.7% (-9.6 to -5.7) |
| Malta | 8902 (8579 to 9227) | 2218.0 (2138.4 to 2296.8) | 10618 (10193 to 10987) | 1904.3 (1833.8 to 1968.6) | -14.1% (-15.8 to -12.2) |
| Monaco | 773 (744 to 800) | 1951.1 (1883.8 to 2018.5) | 896 (862 to 930) | 1804.4 (1739.5 to 1867.5) | -7.5% (-9.3 to -5.7) |
| Netherlands | 365092 (351033 to 377645) | 2172.9 (2091.1 to 2246.5) | 419292 (403345 to 433692) | 1965.5 (1892.7 to 2035.1) | -9.5% (-11.3 to -7.8) |
| Norway | 82969 (80132 to 85727) | 1713.6 (1654.2 to 1769.5) | 102744 (99099 to 106128) | 1593.9 (1540.3 to 1646.5) | -7.0% (-7.5 to -6.5) |
| Portugal | 276492 (266582 to 286221) | 2424.2 (2334.1 to 2506.3) | 286352 (275191 to 296615) | 2081.2 (2001.7 to 2154.8) | -14.1% (-15.8 to -12.5) |
| San Marino | 536 (517 to 556) | 2018.8 (1947.6 to 2091.3) | 749 (720 to 774) | 1851.5 (1782.8 to 1915.0) | -8.3% (-10.2 to -6.4) |
| Spain | 925400 (882993 to 968267) | 2123.3 (2026.8 to 2224.0) | 1088054 (1035189 to 1138966) | 1868.0 (1777.3 to 1956.8) | -12.0% (-14.1 to -10.0) |
| Sweden | 146354 (141010 to 151150) | 1461.5 (1408.1 to 1509.8) | 171693 (165474 to 177426) | 1398.7 (1348.0 to 1446.2) | -4.3% (-5.9 to -2.6) |
| Switzerland | 154246 (148302 to 159433) | 1932.4 (1858.7 to 1998.0) | 196661 (189045 to 203644) | 1798.5 (1732.5 to 1860.9) | -6.9% (-8.7 to -5.0) |
| United Kingdom | 1293646 (1249759 to 1332895) | 1970.2 (1903.8 to 2029.7) | 1414239 (1368451 to 1454700) | 1744.8 (1689.2 to 1795.9) | -11.4% (-11.9 to -11.0) |
| Australasia | 370737 (356706 to 384799) | 1718.3 (1655.2 to 1782.3) | 509347 (490055 to 528245) | 1506.1 (1448.9 to 1560.8) | -12.3% (-13.7 to -10.9) |
| Australia | 303191 (291418 to 315353) | 1685.2 (1621.8 to 1750.6) | 421202 (404638 to 437314) | 1473.1 (1414.2 to 1529.6) | -12.6% (-14.3 to -11.0) |
| New Zealand | 67546 (65159 to 70001) | 1883.8 (1815.6 to 1950.6) | 88145 (84989 to 91137) | 1687.5 (1628.6 to 1744.9) | -10.4% (-11.9 to -8.9) |

Data in parentheses are 95% uncertainty intervals. SDI= Socio-demographic Index.

*Supplementary Table 3*. YLD cases of psoriasis in 1990 and 2019 for both sexes and percentage change of age-standardized rates, by location.

Data in parentheses are 95% uncertainty intervals. SDI= Socio-demographic Index; YLDs= years lived with disability.

| Location | 1990 |  | 2019 |  | Percentage change in age-standardised rates, 1990-2019 |
| --- | --- | --- | --- | --- | --- |
|  | Number | Age-standardized rate  (per 100 000 people) | Number | Age-standardized rate  (per 100 000 people) |  |
| Global | 2732656 (1941601 to 3635507) | 56.7 (40.5 to 75.4) | 3505736 (2504956 to 4638757) | 43.3 (30.9 to 57.4) | -23.6% (-24.2 to -23.0) |
| Low SDI | 123007 (86616 to 163569) | 28.9 (20.5 to 38.2) | 242386 (169711 to 320366) | 25.9 (18.2 to 34.0) | -10.6% (-12.9 to -8.2) |
| Low-middle SDI | 336292 (236529 to 448459) | 35.2 (25.0 to 46.7) | 514982 (364541 to 683588) | 30.3 (21.6 to 40.2) | -13.7% (-15.5 to -11.9) |
| Middle SDI | 555713 (395397 to 743623) | 37.0 (26.5 to 49.3) | 754435 (535544 to 993596) | 29.4 (20.8 to 38.8) | -20.5% (-21.9 to -19.1) |
| High-middle SDI | 742398 (530752 to 982287) | 64.5 (46.1 to 85.3) | 868424 (621609 to 1147162) | 51.1 (36.5 to 67.7) | -20.8% (-21.9 to -19.6) |
| High SDI | 973903 (694638 to 1290072) | 108.1 (76.9 to 143.1) | 1123662 (804184 to 1484634) | 92.3 (65.6 to 122.2) | -14.6% (-15.7 to -13.6) |
| Central Sub-Saharan Africa | 19417 (13731 to 25828) | 43.6 (31.2 to 57.6) | 39139 (27548 to 52169) | 35.6 (25.0 to 47.1) | -18.2% (-23.9 to -12.4) |
| Eastern Sub-Saharan Africa | 23714 (16826 to 31619) | 15.9 (11.4 to 21.0) | 48807 (34514 to 65163) | 14.5 (10.3 to 19.2) | -8.8% (-12.7 to -5.2) |
| Southern Sub-Saharan Africa | 9841 (6936 to 13036) | 21.5 (15.3 to 28.4) | 14553 (10384 to 19199) | 19.2 (13.7 to 25.3) | -11.0% (-15.2 to -6.6) |
| Western Sub-Saharan Africa | 39535 (27806 to 52542) | 25.3 (17.9 to 33.4) | 74188 (52193 to 98677) | 19.5 (13.9 to 25.9) | -22.7% (-24.9 to -20.7) |
| Andean Latin America | 24237 (17073 to 32514) | 74.1 (52.5 to 99.1) | 38808 (27362 to 51290) | 62.1 (44.0 to 82.0) | -16.2% (-20.2 to -11.9) |
| Tropical Latin America | 95504 (67745 to 127451) | 69.5 (49.5 to 92.4) | 157656 (112010 to 207987) | 66.2 (46.9 to 87.6) | -4.6% (-6.9 to -2.5) |
| Central Latin America | 18722 (13176 to 25046) | 13.1 (9.3 to 17.4) | 29285 (20556 to 39034) | 11.6 (8.1 to 15.4) | -11.9% (-15.4 to -8.3) |
| Southern Latin America | 43390 (30637 to 57407) | 89.5 (63.2 to 118.3) | 56128 (39341 to 74462) | 78.0 (54.5 to 103.2) | -12.9% (-17.3 to -8.1) |
| Caribbean | 12703 (8991 to 16865) | 38.8 (27.8 to 51.3) | 17529 (12493 to 23481) | 35.8 (25.5 to 47.9) | -7.6% (-12.2 to -2.9) |
| Central Europe | 62217 (44475 to 82322) | 46.6 (33.2 to 61.7) | 53356 (38034 to 70323) | 38.3 (27.0 to 50.8) | -17.8% (-20.2 to -15.5) |
| Eastern Europe | 103956 (74011 to 136923) | 42.0 (29.7 to 55.3) | 91625 (64980 to 120358) | 36.7 (25.8 to 48.4) | -12.5% (-15.0 to -10.2) |
| North Africa and Middle East | 139162 (98615 to 186438) | 49.0 (34.8 to 65.3) | 211107 (149579 to 279840) | 35.8 (25.5 to 47.3) | -26.9% (-29.0 to -24.6) |
| Central Asia | 30384 (21323 to 40555) | 49.4 (34.8 to 65.7) | 36746 (25998 to 49012) | 39.6 (27.9 to 52.8) | -19.8% (-23.2 to -16.3) |
| South Asia | 287214 (201087 to 383264) | 30.1 (21.3 to 40.0) | 507805 (359434 to 669841) | 28.7 (20.4 to 37.8) | -4.6% (-6.8 to -2.1) |
| Southeast Asia | 51649 (36722 to 69432) | 13.0 (9.3 to 17.5) | 77728 (55264 to 104730) | 11.2 (8.0 to 15.2) | -13.8% (-17.2 to -10.2) |
| East Asia | 577363 (412031 to 771504) | 50.4 (36.1 to 67.2) | 688337 (490650 to 909286) | 38.1 (27.1 to 50.2) | -24.4% (-26.0 to -22.8) |
| Oceania | 1354 (958 to 1820) | 26.3 (18.8 to 35.0) | 2711 (1891 to 3664) | 23.8 (16.7 to 31.9) | -9.3% (-16.9 to -0.7) |
| High-income Asia Pacific | 45309 (32116 to 60623) | 24.0 (17.0 to 32.1) | 52711 (37862 to 69195) | 23.0 (16.4 to 30.7) | -4.1% (-8.4 to 0.1) |
| High-income North America | 326794 (232214 to 432356) | 108.6 (77.0 to 143.5) | 392467 (282948 to 516020) | 92.0 (66.2 to 121.5) | -15.3% (-16.8 to -13.7) |
| Western Europe | 788332 (562709 to 1044219) | 180.0 (127.7 to 239.0) | 871673 (623279 to 1153354) | 163.1 (115.8 to 216.7) | -9.4% (-10.7 to -8.1) |
| Australasia | 31858 (22630 to 41890) | 148.2 (105.1 to 195.6) | 43378 (30992 to 57275) | 129.9 (92.1 to 171.1) | -12.3% (-16.6 to -8.0) |

Data in parentheses are 95% uncertainty intervals. SDI= Socio-demographic Index; YLDs= years lived with disability.

*Supplementary Figure 1*. Age-standardized YLD rate (per 100,000) of psoriasis in 2019 in different SDI countries analyzed for gender.


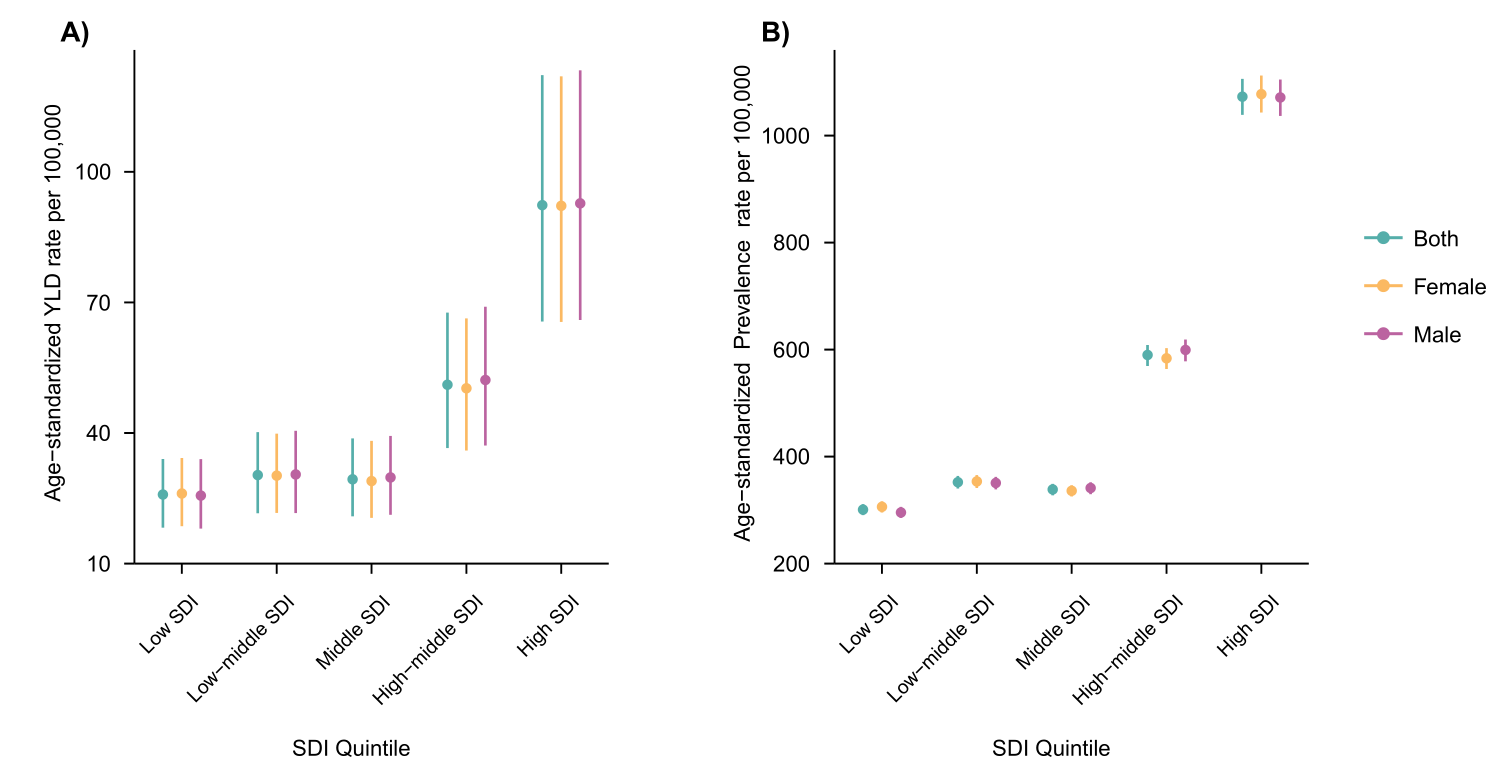


*Supplementary Figure 2*. Change in age-standardized YLD rate of psoriasis by sex and age in all countries (Panel A), in countries with high (Panel B), high-middle (Panel C), middle (Panel D), low-middle (Panel E), and low SDI index (Panel F)


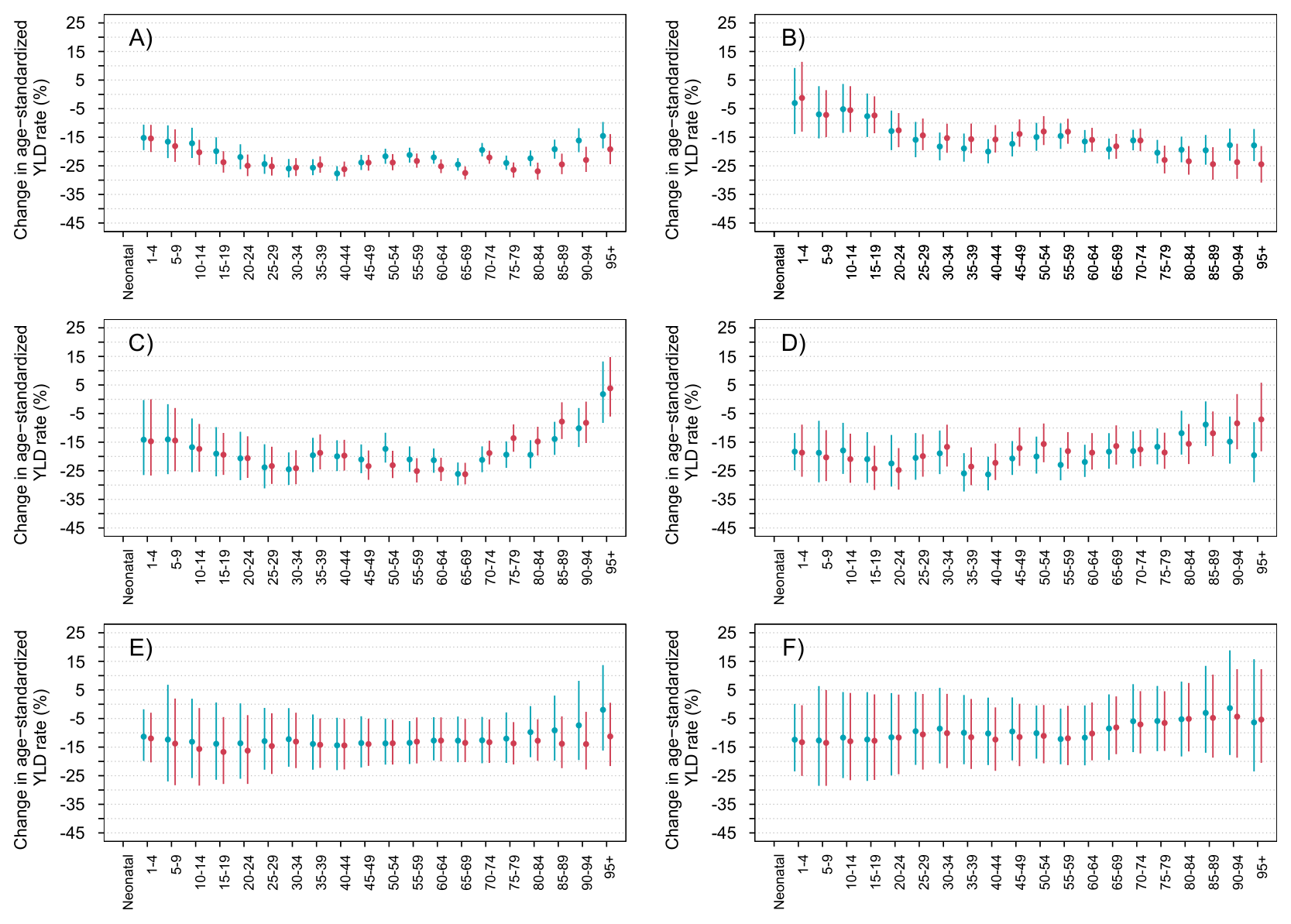


*Supplementary Figure 3*. Age-standardized YLD rate (per 100,000) of psoriasis in 2019 in different GBD super regions analyzed for age-classes.


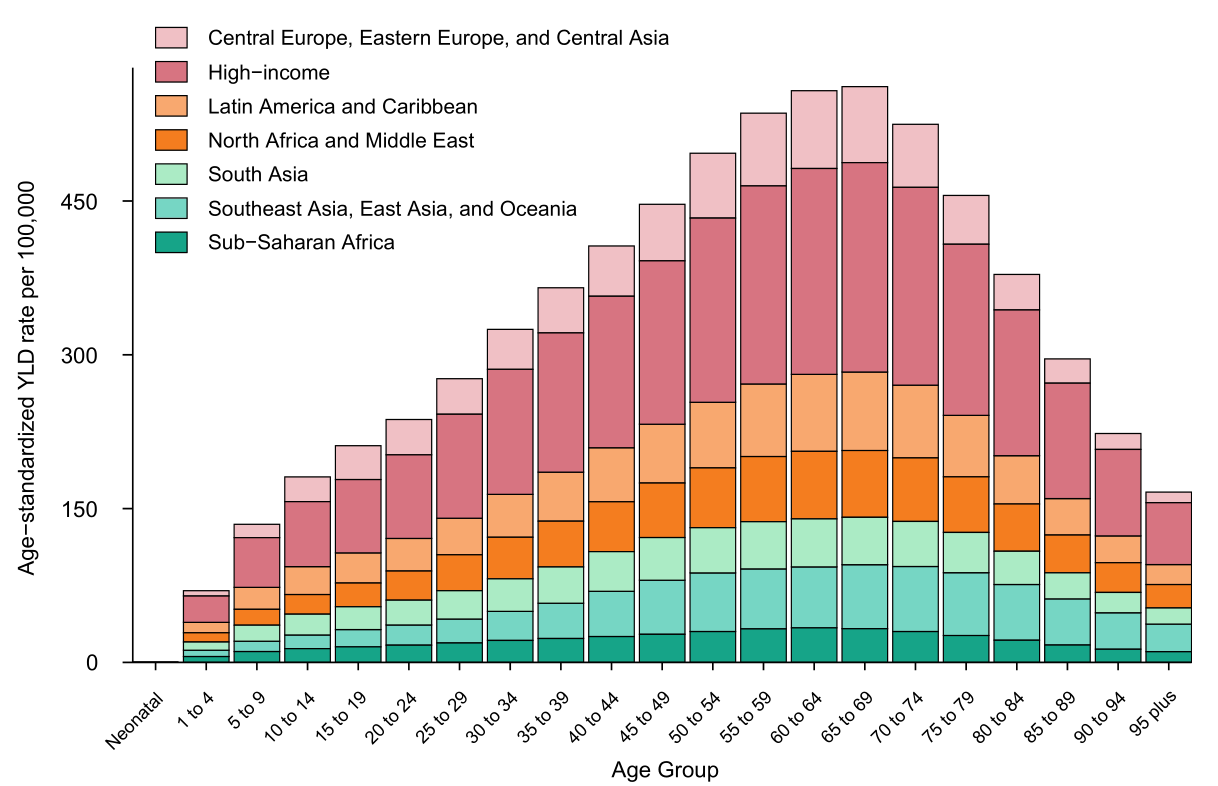


*Supplementary Figure 4*. Age-standardized YLD rate (per 100,000) of psoriasis in 2019 in different GBD super regions analyzed for male, female and combined (both) sexes.


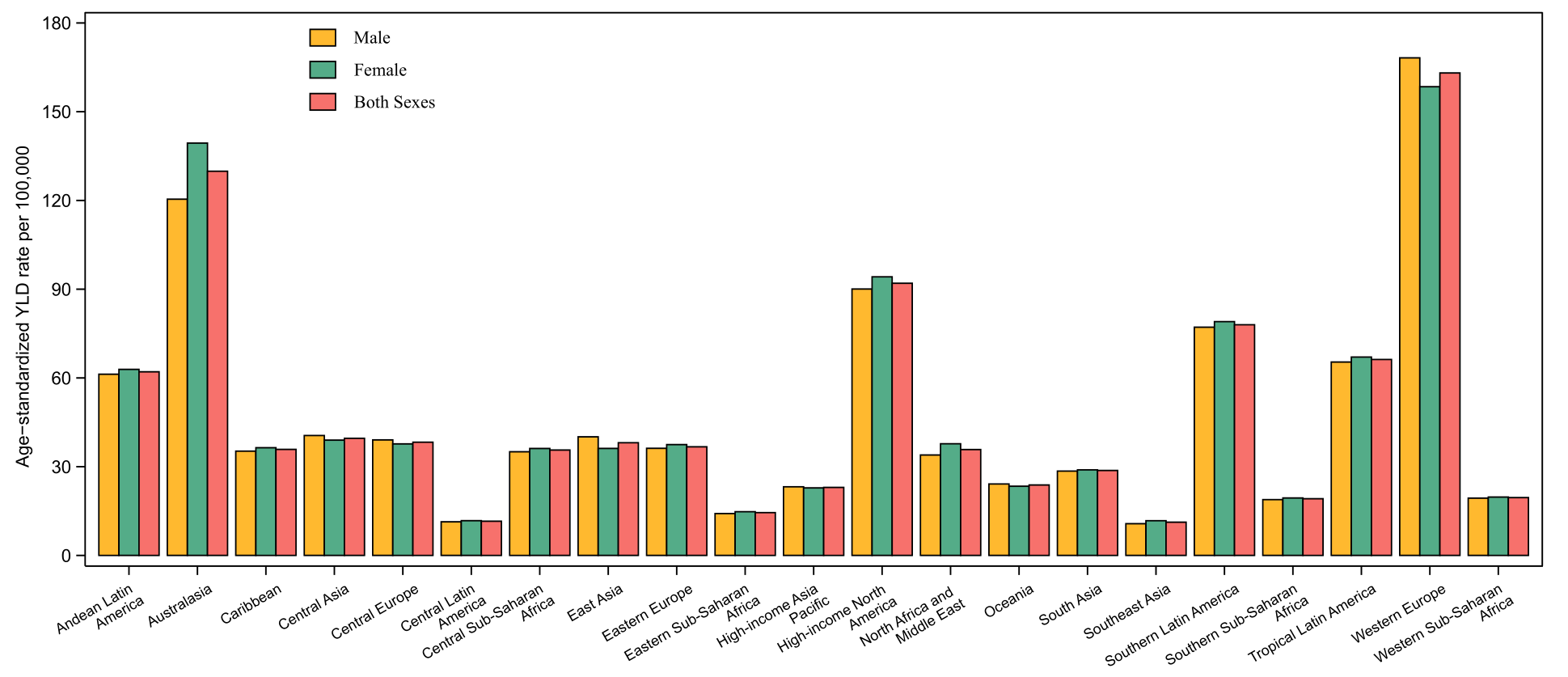

Supplement: Supplementary file 1 [file Data_Sheet_1.docx]
